# A Modular and Customizable CRISPR/Cas Toolkit for Epigenome Editing of *Cis*‐regulatory Modules

**DOI:** 10.1002/advs.202503917

**Published:** 2025-09-29

**Authors:** Lingrui Zhang, Jianxin Fu, Tiandan Long, Chao Zhang, Fuhua Fan, Zhaobo Lang, Jian‐Kang Zhu

**Affiliations:** ^1^ Department of Horticulture and Landscape Architecture Purdue University West Lafayette IN 47907 USA; ^2^ Institute of Advanced Biotechnology, Institute of Homeostatic Medicine, and School of Medicine Southern University of Science and Technology Shenzhen 518055 P. R. China; ^3^ College of Landscape Architecture Zhejiang Agriculture and Forestry University Hangzhou 311300 P. R. China; ^4^ State Key Laboratory of Crop Gene Exploration and Utilization in Southwest China Sichuan Agricultural University Chengdu 625014 P. R. China; ^5^ Institute for Forest Resources and Environment of Guizhou Guizhou University Guiyang 550025 P. R. China

**Keywords:** cis‐regulatory modules, CRISPR/Cas, DNA methylation and demethylation, epigenome editing, synthetic biology

## Abstract

Epigenome and *cis*‐regulome, comprising *cis*‐regulatory elements (CREs) and modules (CRMs), jointly define the architecture of gene regulation. However, the causal mechanisms by which epigenetic marks influence CRM function remain elusive. To address this, modular epigenome editing frameworks, exemplified by dead Cas9‐coupled DNA demethylation (dCd) and DNA methylation (dCm) platforms, are developed for programmable dissection and engineering of CRM activity. The dCd system modulates methylation levels and transcriptional output at CRMs in situ or ex situ, in accordance with CRM‐specific methylation responsiveness, and alters co‐transcriptional RNA processing to yield predictable phenotypic outcomes in plants. These findings underscore the reliability of targeted DNA demethylation. In parallel, the dCm system reconstitutes methylation‐dependent and ‐sensitive CRMs of diverse origins in *Saccharomyces cerevisiae*, a species devoid of native DNA methylation, enabling causal dissection of epigenetic regulation and revealing cross‐species portability. This system further uncovers crosstalk between DNA methylation and chromatin modifications, and enables logic‐gated control of endogenous genes through CRM engineering. Incorporation of optogenetic and temperature‐sensitive anti‐CRISPR inhibitors confers tunable, reversible regulation, proposing dCm as a foundation for input‐responsive synthetic epigenome editors. Together, these frameworks provide a versatile platform to decode and reprogram *cis*‐regulatory epigenetic logic, with broad applications in trait design and synthetic biology.

## Graphical abstract

Epigenome editing surpasses genome editing in gene regulation. Modular epigenome editing frameworks are developed to interrogate *cis*‐regulome, enabling tunable reprogramming of *cis*‐elements, uncovering their epigenetic responsiveness and cross‐species portability. Informed by these new insights, a plant‐specific “methylstat” powers engineering in non‐DNA‐methylating yeast, achieving logic‐gated epigenetic control of endogenous genes and opening a new dimension in circuit design for synthetic biology.

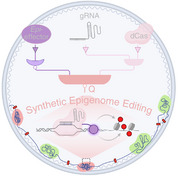

## 1 Introduction

A fundamental tenet in biology for any organism is the regulation of gene expression. At the heart of this process are non‐coding *cis*‐regulatory sequences, which contain essential information that determines the patterns and levels of gene expression.^[^ ^1^, ^2^, ^3^ ^]^ These sequences constitute the *cis*‐regulome, encompassing *cis*‐regulatory elements (CREs), which are primary DNA units that interact with *trans*‐acting factors such as transcription factors (TFs), and *cis*‐regulatory modules (CRMs), which are assemblies of CREs functioning at the level of promoters, enhancers, silencers, insulators, and multifunctional switchable sequences.^[^ ^1^, ^4^ ^]^

The *cis*‐regulome is intricately intertwined with the epigenome, in that CRMs regulate their target genes through epigenetic signatures.^[^ ^5^, ^6^ ^]^ CRMs are associated with active histone modifications that increase chromatin accessibility and facilitate transcription, whereas repressive marks reduce accessibility and establish silenced states.^[^ ^1^, ^6^, ^7^, ^8^, ^9^ ^]^ In addition to histone modifications, DNA methylation serves as another epigenetic mechanism that dictates CRM functionality. In the model organism *Arabidopsis thaliana (A. thaliana)*, a significant proportion of CREs are genic (overlapping a gene) or proximal (within 2 kb of a gene),^[^ ^2^ ^]^ in contrast to maize, which harbors numerous distal CREs across its large and complex genome.^[^ ^8^, ^10^, ^11^ ^]^ This contrast illustrates how genome size can shape the spatial distribution of CREs. Notwithstanding variation in genomic positioning, the majority of CREs in *A. thaliana* exhibit low levels of cytosine DNA methylation, a trend consistent across multiple species.^[^ ^1^, ^2^, ^12^ ^]^ Functionally, proximal DNA methylation often suppresses gene expression, and many *A. thaliana* TFs are binding‐incompetent for methylated cytosines.^[^ ^13^, ^14^, ^15^ ^]^ A well‐characterized example is the widely used *35S* promoter from cauliflower mosaic virus (CaMV), which is highly susceptible to DNA methylation‐mediated repression, resulting in transgene silencing.^[^ ^16^, ^17^, ^18^ ^]^ Augmenting these observations, active DNA demethylation at CRMs has been shown to enhance gene expression in specific developmental processes, such as fruit ripening in tomato, and in plant responses to stress conditions, such as those triggered by pathogen attacks.^[^ ^19^, ^20^, ^21^, ^22^, ^23^, ^24^, ^25^ ^]^

However, there are notable exceptions where DNA methylation at proximal CRMs is required for gene expression, likely mediated by a DNA methylation reader complex.^[^ ^26^, ^27^, ^28^, ^29^ ^]^ An illustrative example is the transposon‐related *cis*‐sequence known as the extended DNA methylation monitoring sequence (eMEMS), located in the promoter of the *REPRESSOR OF SILENCING 1* (*ROS1*) demethylase gene, where DNA methylation is essential for *ROS1* expression.^[^ ^18^, ^28^, ^30^ ^]^ This marriage of eMEMS with DNA methylation signature establishes a dynamic and sophisticated “methylstat” circuit in plants, finely tuning DNA methylation homeostasis and potentially enabling adaptive transcriptional responses to endogenous and environmental cues.^[^ ^31^, ^32^ ^]^ While DNA methylation is best known for its role in regulating transcriptional activity, it also contributes to co‐transcriptional RNA processing, notably distal polyadenylation. A representative case is *INCREASED IN BONSAI METHYLATION 1* (*IBM1*), where DNA methylation within a large intron, alongside H3K9me2, is indispensable for generating the long *IBM1* transcript required for active isoform production.^[^ ^17^, ^33^, ^34^, ^35^ ^]^ By dynamically regulating its own transcript variants through intronic epigenetic modifications, IBM1 plays a pivotal role in overseeing genic DNA methylation and global H3K9me2 levels, ensuring transcriptional homeostasis across the genome.^[^ ^36^ ^]^ Together, these findings underscore the multifaceted roles of DNA methylation in shaping gene regulatory landscapes, dynamically decorating CRMs and orchestrating transcriptional precision across plant genomes.

Yet despite growing insights into CRM‐epigenome interactions, the regulatory functions of most CRMs in response to epigenetic cues remain poorly understood.^[^ ^2^, ^37^ ^]^ The ongoing discovery of CREs and the assembly of novel, particularly uncustomary, CRMs further underscore the urgent need to determine how these emerging CRMs function within epigenetic landscapes.^[^ ^1^, ^4^, ^37^ ^]^ However, compared to the breakthrough technologies that have accelerated CRM discovery, functional validation strategies have significantly lagged behind, thereby impeding our comprehension of *cis*‐regulome and their potential for engineering genetic circuits in synthetic biology.^[^ ^1^, ^4^, ^15^, ^37^ ^]^ Clustered Regularly Interspaced Short Palindromic Repeat (CRISPR)/CRISPR‐associated protein (Cas) systems, originating from prokaryotic defense mechanisms, have evolved from their initial role in site‐specific mutagenesis,^[^ ^38^, ^39^ ^]^ into versatile platforms, including present‐day precision genome editing^[^ ^40^, ^41^ ^]^ and targeted epigenome editing.^[^ ^42^, ^43^ ^]^ In mammalian systems, deactivated Cas (dCas) combined with epigenetic effectors has been broadly applied to modify the epigenetic states of diverse regulatory elements, including those lacking canonical CpG islands,^[^ ^42^, ^43^, ^44^, ^45^, ^46^, ^47^ ^]^ and systematic frameworks have also been developed to enable programmable and scalable CRM manipulation for precise transcriptional control, with ongoing efforts aimed at improving precision and efficiency.^[^ ^43^, ^48^, ^49^, ^50^ ^]^ CRISPR/dCas‐based epigenome editing has also been demonstrated in plants.^[^ ^51^, ^52^, ^53^, ^54^, ^55^, ^56^, ^57^, ^58^ ^]^ However, its effectiveness has only been validated for a few regulatory elements. These primarily include naturally occurring epialleles, such as the hypomethylated versions of *FLOWERING WAGENINGEN* (*FWA*) and *SUPERMAN*, which, while offering clear phenotypic readouts, limit generalizability to broader regulatory contexts.^[^ ^51^, ^53^, ^56^, ^58^, ^59^, ^60^ ^]^ Moreover, current tools are often structurally rigid and lack modular design, constraining their adaptability to logic‐based editing architectures and their evolution into tunable epigenetic editors.^[^ ^43^, ^61^, ^62^ ^]^ Most critically, existing approaches are confined to in situ genomic contexts and are incapable of reconstituting CRMs or interrogating their function in synthetic settings, thereby diminishing efforts to assess CRM portability and robustness across chromatin environments and species boundaries.

Herein, we devised two CRISPR/dCas‐based epigenome editing frameworks, instantiated as dCas9‐coupled DNA demethylation (dCd) and dCas9‐coupled DNA methylation (dCm) systems. Designed for modularity and adaptability, these frameworks enable customizable reconfiguration. The resulting dCd and dCm platforms were validated for manipulating diverse CRMs both in situ and across distinct chromatin contexts, supporting functional studies of CRM transferability. Tunable dCm was demonstrated via optogenetic and temperature‐sensitive modules, and logic‐based control of endogenous genes was achieved through dCm‐mediated methylation, in DNA methylation‐deficient hosts. Collectively, these advances offer a foundation for dissecting the *cis*‐regulome and building epigenetically controlled genetic circuits in synthetic biology.

## 2 Results

### 2.1 Frameworks on the Construction of Epigenome Editing Toolkits

**Figure 1:**
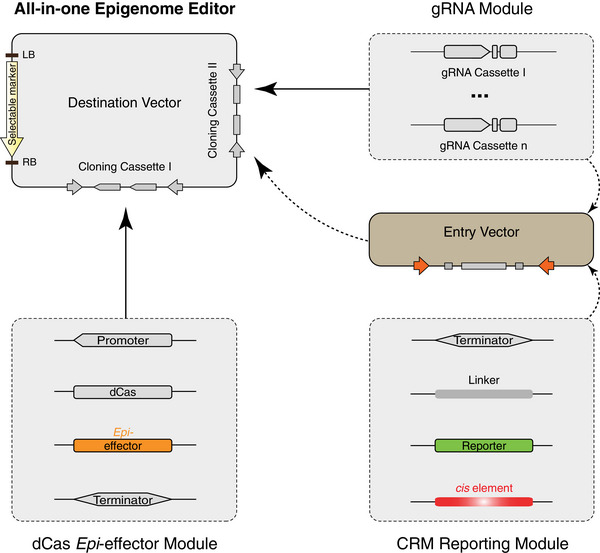
Conceptual Framework for Modular Epigenome Editing in Planta. The dCas *Epi*‐effector and gRNA modules are integrated into a destination vector carrying a selectable marker via distinct cloning cassettes (solid arrowheads), enabling in situ epigenome editing. Alternatively, the gRNA and CRM reporter modules can be pre‐assembled into an entry vector (dashed arrowheads), subsequently forming an all‐in‐one construct for targeted CRM epigenome editing. Each module comprises interchangeable blocks, allowing programmable, reconfigurable assembly, such as flexible gRNA cassette tuning (ellipses). A detailed schematic of the complete assembly framework is provided in Figure S1, Supporting Information.

**Figure 2:**
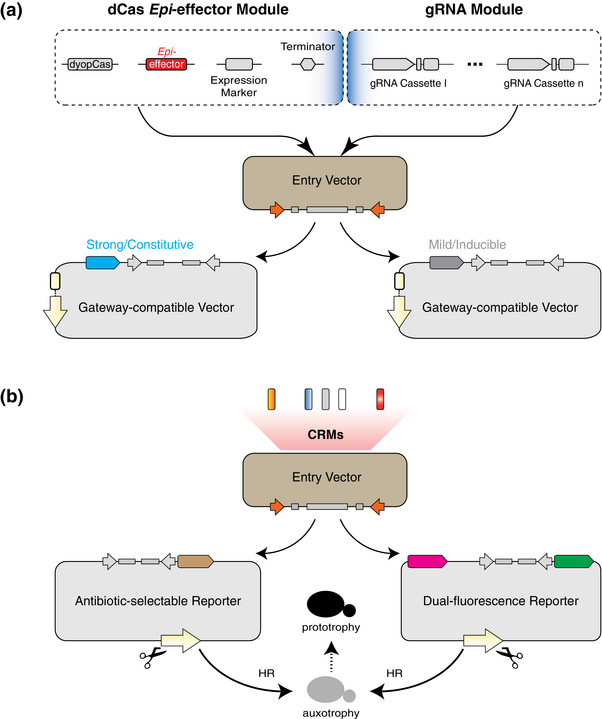
Conceptual Framework for Modular Epigenome Editing in *S. cerevisiae*. a) The dyopCas *Epi*‐effector and gRNA modules are pre‐assembled into an entry vector using interchangeable parts (e.g., *Epi*‐effectors, selectable expression markers, and variable gRNA cassettes denoted by ellipses). These assemblies are recombined into Gateway‐compatible expression vectors with tunable promoters supporting either constitutive or inducible expression, and can be deployed in genomic or episomal contexts via distinct replication origins and auxotrophic selection markers (yellow highlights). b) CRMs of interest are cloned into an entry vector and recombined into destination vectors configured for antibiotic resistance or dual‐fluorescence reporter readouts. Reporter constructs are integrated at auxotrophic loci via HR, yielding stable transformants through prototrophic rescue, enabling CRM‐targeted epigenome editing. A full schematic of the assembly pipeline is provided in Figure S2, Supporting Information.

To equip the epigenome editing toolkits with versatility and multiple adaptability, we developed multi‐tiered frameworks that integrate independent blocks into modular assemblies (**Figures** **1** and 2; Figures S1 and S2, Supporting Information). By abstracting system design into reconfigurable tiers, our framework overcomes the rigidity of monolithic constructs. This architecture supports part‐level customization, module‐level interchangeability, and system‐level scalability, allowing editing strategies to be iteratively evolved and adapted across species with minimal redesign. The first framework is tailored for use in planta, where the CRISPR/dCas epigenome effector (*Epi*‐effector) module can be adapted to distinct epigenetic marks, and the guide RNA (gRNA) module can deliver up to four gRNAs for fine‐tuned targeting (**Figure** **1**; Figure S1, Supporting Information). The unique combination of a modular CRM reporting platform, capable of accommodating distinct reporters and diverse CRMs, enables an all‐in‐one assembly for CRM epigenome editing in synthetic contexts (Figure 1; Figure S1 and Notes S1–S3, Supporting Information). Beyond epigenome editing, the framework may be extended to transcriptional regulation via chromatin remodelers and *trans*‐acting TFs.^[^ ^56^ ^]^ In the following section, we demonstrate the flexibility of this framework in assembling epigenome editors with diverse *Epi*‐effectors and gRNA combinations, and validate its efficacy in both in situ and synthetic‐context epigenome editing of multiple CRMs (see Section 2.2).

The second framework is specifically designed for utilizing the non‐DNA methylation *Saccharomyces cerevisiae* (*S. cerevisiae*) as a model, enabling CRM testing and dCm deployment without confounding native DNA methylation (**Figure** **2**; Figure S2, Supporting Information).^[^ ^63^, ^64^ ^]^ Similar to the plant modules, the CRISPR/dCas *Epi*‐effector module, built using dead yeast codon‐optimized Cas (dyopCas), and the gRNA module are highly adaptable. By introducing selectable expression markers, such as fluorescent proteins (FPs) or antibiotic resistance genes, yeast cells expressing the *Epi*‐effector can be enriched, minimizing false positives that often occur in yeast‐based assays (Figure 2a; Figure S2a and Note S4, Supporting Information). Pre‐assembled modules in an entry vector are compatible with Gateway‐based constitutive or inducible yeast expression systems, offering different expression amplitudes and enabling the construction of input‐responsive synthetic epigenome editing circuits. The CRM reporting systems include a high‐sensitivity antibiotic‐based module and a contrasting dual‐fluorescence (DF)‐based module, both integrated into the yeast genome via homologous recombination (HR) and auxotrophic selection (Figure 2b; Figure S2b, Supporting Information). This design supports targeted epigenome editing of CRMs from diverse origins in a clean and controlled synthetic context, thereby uncovering the causal roles of epigenetic modifications in CRM activities and their functional portability across species. Our validation confirms that this framework effectively enables epigenome editing, faithfully replicates CRM functionalities observed in their native environments, and supports epigenome editing‐mediated, logic‐gated control of endogenous gene expression in yeast (see Section 2.3).

### 2.2 CRISPR/dCas9‐based Targeted DNA Demethylation Editing of CRMs in Planta

**Figure 3:**
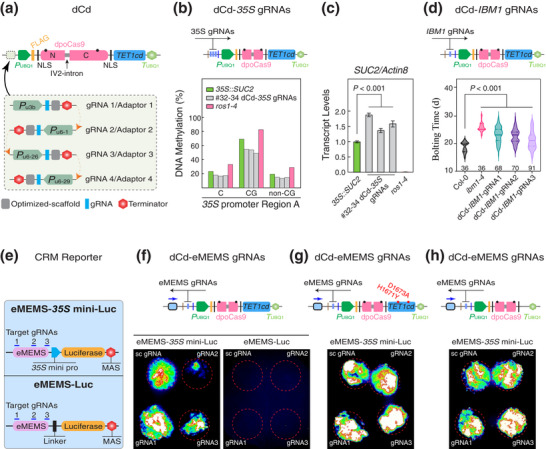
The dCd System Enables Epigenome Editing of CRMs in Planta. a) Schematic of the TET1cd‐centered dCd system and gRNA module. Black dots indicate mutations introduced into the N‐terminal (N) and C‐terminal (C) regions of Cas9. Dashed orange arrowheads indicate the assembly orientation of four gRNA cassettes or adaptors (Figure S1 and Notes S1 and S2, Supporting Information). b) CG and non‐CG DNA methylation levels within Region A of the CaMV *35S* promoter, which drives *SUC2*, were analyzed in three independent transgenic lines expressing dCd‐*35S* gRNAs and in control lines. The *35S* promoter regions and gRNA target sites are shown in Figure S3a, Supporting Information. The *ros1‐4* mutant is in the *35S::SUC2* background. c) *SUC2* expression values are means ± SD from three biological replicates, normalized to *Actin 8* and expressed as fold‐change relative to *35S::SUC2* controls (set to 1) across genotypes. d) Bolting time for dCd‐*IBM1* gRNA transgenic and control lines is shown as violin plots. Numbers above each genotype indicate the number of T1 plants analyzed. Three *IBM1*‐targeting gRNAs were used to target the sites shown in Figure S3d, Supporting Information. e) Schematic of the CRM reporting system used to evaluate eMEMS activity, configured with or without the *35S* mini pro (Figure S1 and Note S3, Supporting Information). gRNA target sites in eMEMS are numbered and marked with short blue lines. CRM modules are integrated into the dCd system at the position marked by the empty rectangle. f–h) Reporter regulation by the dCd system using either target or sc gRNAs, under three TET1cd configurations: wild‐type (f), mutated (g, red dots), and deleted (h). Black and blue arrowheads indicate the orientations of gRNA cassettes and reporting modules, respectively. Statistical significance was determined using an unpaired two‐tailed Student's test. Additional replicates and primer sequences for gRNAs are provided in Figure S4 and Table S1, Supporting Information.

Based on the construction schema (Figure 1; Figure S1, Supporting Information), we first established the dCd system. In this system, a dead plant codon‐optimized *Streptococcus pyogenes* (*S. pyogenes*) Cas9 (dpoCas9) is N‐terminally fused to the catalytic domain of the human 5‐methylcytosine dioxygenase TEN‐ELEVEN TRANSLOCATION 1 (TET1cd), forming a fusion protein expressed under the *A. thaliana UBIQUITIN 1* (*UBQ1*) promoter (**Figure** **3**a; Figure S1, Table S2, and Note S1, Supporting Information). gRNAs or adaptors can be flexibly configured according to specific targets or experimental requirements (Figures 1 and 3a; Figure S1, Supporting Information). Synchronously incorporating the dCd system with three gRNAs, which target the distal region (Region A) of the CaMV *35S* promoter controlling the expression of the *SUCROSE TRANSPORTER 2* transgene (*35S::SUC2*), led to reduced DNA methylation in three independent lines relative to the original *35S::SUC2* plants (Figure 3b; Figure S3a, Supporting Information). In contrast, no substantial change was observed in the untargeted proximal region (Region B; Figure S3a,b, Supporting Information). Consistent with the reduced DNA methylation, *SUC2* expression was significantly increased in the dCd transgenic lines compared to the original *35S::SUC2* plants (*P* < 0.001; Figure 3c; Figure S3c, Supporting Information). As controls, *ros1‐4* mutants in the *35S::SUC2* background exhibited increased DNA methylation in Region A, resulting in suppressed *SUC2* expression (Figure 3c; Figure S3c, Supporting Information). These observations are consistent with previous findings on the regulation of the *35S* promoter and further underscore the critical role of DNA methylation in transgene silencing.^[^ ^16^, ^17^, ^18^, ^35^ ^]^

Next, we separately introduced three gRNAs, which target the long intron within the *IBM1* locus, into the dCd system (Figure 3d; Figure S3d, Supporting Information). Similar to the *ibm1‐4* mutants, T1 transgenic lines harboring the *IBM1*‐targeting dCd system bolted significantly later than the Col‐0 control (*p* < 0.001; Figure 3d). Although the DNA methylation was not analyzed in this case, the result suggested that the dCd system reduced the *IBM1* intronic DNA methylation, which in turn caused transcript mis‐processing and thus reduced levels of long, functional IBM1 isoform.^[^ ^17^, ^33^, ^34^, ^35^ ^]^ Taken together, these findings indicate that the developed dCd system can effectively modulate the epigenetic state of regulatory sequences, thereby altering gene expression, transcript processing, and associated phenotype outcomes.

To investigate CRM functionality outside the native genomic context, we equipped the dCd system with a CRM reporting module (Figure 1; Figure S1, Supporting Information) and evaluated its ability to assess the regulatory activity of tested CRMs in a synthetic and controlled setting. We selected eMEMS from the Col‐0 ecotype for its well‐characterized dependence on DNA methylation to regulate *ROS1* expression.^[^ ^18^, ^30^ ^]^ We positioned eMEMS upstream of the luciferase (Luc) reporter, either with or without the *35S* minimal promoter (*35S* mini pro; Figures 1 and 3e; Note S3, Supporting Information). In the absence of *35S* mini pro, no luminescent signal was detected in the *Nicotiana benthamiana* (*N. benthamiana*) transient expression platform, irrespective of gRNAs (Figure 3e,f; Figure S4b, Supporting Information). By contrast, in the presence of *35S* mini pro, eMEMS successfully activated the Luc reporter (Figure 3e,f; Figure S4a,b, Supporting Information). Notably, relative to the scrambled gRNA (sc gRNA), reporter activation was markedly suppressed by the dCd system when employing gRNA2, which targets the middle site of eMEMS, though gRNA1 and gRNA3, targeting the termini, had no comparable effect (Figure 3e,f; Figure S4a,b and Note S3, Supporting Information).

The specific effect observed with gRNA2, but not with gRNA1 or gRNA3, suggests that dCd‐mediated CRM editing is confined to a narrow region surrounding the gRNA target site. This interpretation is consistent with prior findings showing that dCas9‐TET1cd acts within a small window centered on the binding site.^[^ ^46^ ^]^ To validate that the reduced luminescent signal was indeed caused by DNA demethylation, we either inactivated TET1cd through previously characterized catalytic mutations or removed TET1cd entirely from the dCd system.^[^ ^65^ ^]^ In both cases, despite the presence of gRNA2, the suppressive effect on reporter activity was abolished (Figure 3g,h; Figure S4c and Table S2, Supporting Information). These findings indicate that the suppression of eMEMS‐mediated gene expression by dCd is strictly dependent on the demethylation function of TET1cd. This positive link between DNA methylation and gene expression is private to a few known loci, including eMEMS.^[^ ^18^, ^28^, ^29^, ^30^ ^]^ In accordance with this notion, a *cis*‐element from *LEUCYL AMINOPEPTIDASE 1* (*LAP1*), which is not regulated by DNA methylation, drove luminescence irrespective of gRNAs (Figure S4d, Supporting Information). These findings demonstrate that the dCd system can reliably distinguish CRMs with differential dependence on DNA methylation in regulating gene expression.

### 2.3 CRISPR/dCas9‐based Targeted DNA Methylation System for Illuminating CRMs in Non‐DNA Methylation Contexts

Building on the construction framework (Figure 1; Figure S1, Supporting Information), we substituted TET1cd with *A. thaliana* DOMAINS REARRANGED METHYLTRANSFERASES 2 (DRM2) or its catalytic domain (DRM2cd) to generate the dCm system for targeted DNA methylation in planta (Table S2, Supporting Information). In the *N. benthamiana* expression platform, however, neither DRM2 nor DRM2cd produced a noticeable enhancement of eMEMS‐mediated reporter activity in the presence of gRNA2 compared to sc gRNAs (Figure S5, Supporting Information). This outcome likely reflects pre‐existing DNA methylation mechanisms in *N. benthamiana*, which may have saturated the eMEMS‐based reporting system and masked any additional effects of targeted methylation (see Discussion below).

**Figure 4:**
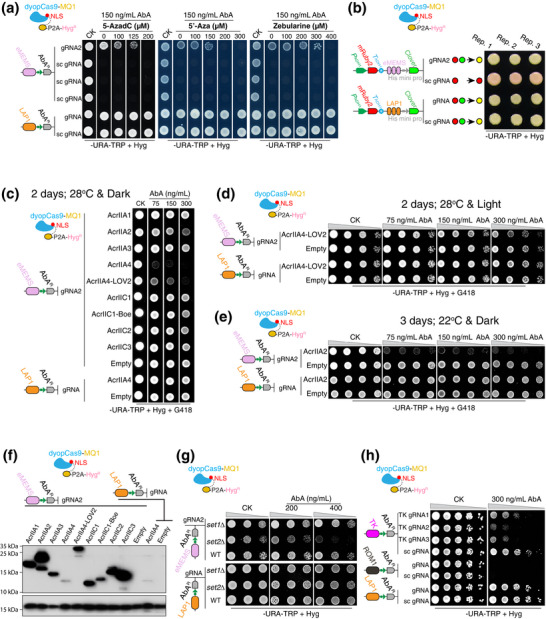
The dCm System Enables Epigenome Editing of CRMs in Heterologous Platforms. a) Schematic and functional evaluation of the MQ1‐centered dCm system (Figure 2; Figure S2 and Notes S4 and S5, Supporting Information). The system was tested for activation of the eMEMS‐coupled *AbA^R^* reporter in the Y1H Gold strain using target gRNA2 (Figure 3e), sc gRNAs, and three DNA methylation inhibitors. Additional strain‐specific results are provided in Figures S6–S8, Supporting Information. b) Evaluation of eMEMS(3x)‐driven *Clover* expression in the DF system. Red and green circles represent *mRuby2* and *Clover* expression, respectively; yellow circles indicate merged fluorescence. Three replicates per group are shown. c–e) Assessment of eMEMS activity in W303a strains under anti‐CRISPR interference across the indicated conditions. Additional results are provided in Figures S10 and S11, Supporting Information. f) Anti‐CRISPR inhibitor expression was analyzed by immunoblotting with anti‐HA antibody; GAPDH was used as a loading control (lower panel). Protein extracts were obtained from yeast cultured under the same conditions as in (c), without AbA supplementation. g) eMEMS activity was assessed in W303a strains expressing dCm under the *ROX3* promoter in WT and indicated mutant backgrounds. h) Experimental configuration for testing *TK* promoter regulation by the dCm system with gRNAs. ROM1 and LAP1 *cis*‐fragments were used as negative and positive controls, respectively (Tables S1 and S2, Supporting Information). Selection conditions were indicated below each panel. Data represent single colonies, diluted or undiluted, with consistent outcomes observed in six out of eight colonies for each of the two independent experiments.

To overcome interference from native DNA methylation, we implemented the dCm system within the second framework using *S. cerevisiae*, a non‐DNA methylation model organism, to functionally interrogate CRMs derived from higher organisms (Figure 2; Figure S2, Supporting Information). In this yeast‐adaptable dCm system, an engineered DNA methyltransferase (MQ1^Q147L^, hereafter referred to as MQ1) was fused to the C‐terminus of dyopCas9 derived from *S. pyogenes*, which was further linked, via a P2A peptide, to a Hygromycin resistance (Hyg^R^) gene for selection (Table S2 and Note S4, Supporting Information).^[^ ^66^, ^67^ ^]^ Using this system, the chimeric eMEMS‐Aureobasidin A resistance (*AbA^R^*) gene reporter was activated in three yeast strains, hinging on the presence of eMEMS gRNA2 but not sc gRNAs; moreover, this activation was reliant on DNA methylation, as three inhibitors effectively suppressed reporter activation across all tested strains (**Figure** **4**a; Figures S6–S8, Supporting Information). Notably, the inhibitors exhibited distinct potencies relative to one another across all strains; for instance, Zebularine required higher concentrations to suppress the eMEMS‐*AbA^R^* reporter activation than the other inhibitors (Figure 4a; Figures S6–S8, Supporting Information). Alongside the differentiated responses of yeast strains to the inhibitors (Figure 4a; Figures S6–S8, Supporting Information), our results demonstrate the utility of the dCm system in evaluating the stoichiometric characteristics of DNA methylation inhibitors and in capturing strain‐specific nuances in methylation‐dependent transcriptional regulation. In agreement with findings from the dCd system (Figure 3e–h; Figure S4, Supporting Information), dCm‐mediated activation of the eMEMS‐*AbA^R^* reporter in yeast reaffirmed its dependency on DNA methylation for gene expression, whereas the LAP1‐chimeric reporter remained constitutively active irrespective of gRNA identity or inhibitor treatment, consistent with its independence from DNA methylation (Figure 4a; Figures S6–S8, Supporting Information). To further validate dCm‐mediated activation of eMEMS, we extended our analysis to orthologous eMEMS sequences from the C24 ecotype and *Arabidopsis lyrata*.^[^ ^18^, ^30^ ^]^ Consistent with Col‐0 eMEMS, both variants effectively enabled reporter expression in the presence of the corresponding target gRNA compared to sc gRNA or negative control, albeit with reduced potency for the *A. lyrata* variant (Figure S9, Supporting Information). Collectively, these findings demonstrate that the dCm system enables ex situ epigenetic manipulation of eMEMS, recapitulating its native methylation‐dependent role in gene expression.

To ascertain whether dCm‐mediated eMEMS activation can extend to a different reporter, we integrated eMEMS into an alternative DF reporter system (Figure S2b and Table S2, Supporting Information). In this system, eMEMS activity manipulated by the dCm system is monitored via Clover fluorescence (green), while *mRuby2* (red) is constitutively expressed as an internal control to contrast reporter activation (Figures 2b and 4b). Results demonstrated that in the presence of eMEMS gRNA2, the dCm system activated the chimeric eMEMS‐*Clover* reporter, resulting in yellow cells when merged with mRuby2 red fluorescence, similar to the LAP1‐*Clover* positive control, which was independent of gRNA targeting as well (Figure 4b). By contrast, cells expressing sc gRNA exhibited only red fluorescence, indicating no eMEMS‐*Clover* activation (Figure 4b). These results indicate that dCm‐mediated activation of eMEMS is transferable across different reporters.

To affirm that eMEMS‐coupled reporter activation is specifically driven by the dCm system, we evaluated a panel of anti‐CRISPR proteins, including four inhibitors of type II‐A CRISPR‐Cas9 (AcrIIA1‐4), derived from *Listeria monocytogenes* (*L. monocytogenes*) prophages, and three inhibitors (AcrIIC1‐3) targeting type II‐C Cas9 orthologs. Among these, AcrIIA4 displayed a strong inhibitory effect on dCm‐mediated activation of the eMEMS‐*AbA^R^* across different yeast strains (Figure 4c; Figures S10 and S11a, Supporting Information). A chimeric AcrIIA4‐LOV2 fusion, engineered by inserting the photosensor LOV2 domain into AcrIIA4,^[^ ^68^ ^]^ inhibited eMEMS activation in the dark but not under light (Figure 4c,d; Figure S11a,b, Supporting Information). This aligns with previous findings demonstrating that light‐induced conformational changes in the LOV2 domain disrupt the functional folding of AcrIIA4, thereby impairing its ability to inhibit Cas9 activity or trap dCas9.^[^ ^68^ ^]^

At a lower temperature (22 °C), another type II‐A CRISPR‐Cas9 inhibitor, AcrIIA2, displayed a marked inhibitory effect, which was less pronounced at a higher temperature (28 °C; Figure 4c,e; Figures S10 and S11, Supporting Information). In contrast, other anti‐CRISPR proteins tested failed to block the activation of the eMEMS‐*AbA^R^* reporter by the dCm system, despite being expressed in yeast cells (Figure 4c,f; Figures S10 and S11, Supporting Information). These observations are consistent with previous findings that only AcrIIA4 and AcrIIA2 exhibit broad‐spectrum activity in abrogating target binding of *S. pyogenes* Cas9 or dCas9, which shares 53% amino acid identity with *L. monocytogenes* Cas9, and that AcrIIA2 is more temperature‐sensitive than AcrIIA4.^[^ ^69^, ^70^ ^]^ Our experiments indeed revealed that AcrIIA2 was unstable, while AcrIIA4 was barely detectable, likely due to its competitive binding to *S. pyogenes* Cas9 (Figure 4f).^[^ ^70^ ^]^ Given that these inhibitors had no effect on dCm‐independent activation in the LAP1 control groups (Figure 4c–e; Figures S9 and S10, Supporting Information), our results confirm the specificity of dCm‐mediated, DNA methylation‐dependent activation in eMEMS. Moreover, our findings highlight the capacity of the dCm system for tunable epigenome editing of CRMs, and provide a convenient strategy to assess the efficacy of anti‐CRISPR inhibitors.

To determine whether and how dCm‐deposited DNA methylation interacts with chromatin modifications in gene activation, we generated *set1Δ* (deficient in H3K4 methylation) and *set2Δ* (deficient in H3K36me3) yeast mutant strains.^[^ ^71^, ^72^, ^73^ ^]^ Our results showed that, in both tested backgrounds, the activation of the chimeric eMEMS reporter by the dCm system was severely inhibited in *set2Δ* mutants relative to wild‐type (WT) strains, whereas reporter activation remained unaffected in *set1Δ* mutants (Figure 4g; Figure S12, Supporting Information). This suggests that H3K36me3, rather than H3K4 methylation, is necessary for dCm‐mediated gene activation. The conclusion was further validated in *swd1Δ* mutants, which are also deficient in H3K4 methylation,^[^ ^73^ ^]^ showing no effect on eMEMS activity (Figure S12, Supporting Information). Notably, LAP1‐driven expression remained unchanged across WT and mutant backgrounds (Figure 4g), confirming that the observed effects are specific to the dCm‐mediated process. Together, these findings suggest that dCm‐induced DNA methylation promotes gene expression in a Set2‐dependent but Set1‐independent manner. Considering the roles of these chromatin modifications, it is plausible that the dCm‐introduced DNA methylation in eMEMS may either override H3K4 methylation‐mediated gene priming or directly activate gene expression,^[^ ^71^ ^]^ while H3K36me3 may be necessary for promoting an open chromatin state that is conducive for transcription in the context of DNA methylation.^[^ ^72^ ^]^

To validate the generality of this dCm system, we applied it to epigenetically manipulate CRMs derived from other organisms or biological entities. Placing the promoter of herpes simplex virus type 1 (HSV1) *THYMIDINE KINASE* (*TK*) upstream of *AbA^R^* supported yeast survival in the presence of AbA; however, yeast growth was substantially retarded by the dCm system in the presence of target TK gRNAs (Figure 4h). This result aligns with previous findings that the promoter of HSV1 *TK* is sensitive to DNA methylation.^[^ ^74^ ^]^ Next, we examined two gene clusters in tomato (*Solanum lycopersicum*) previously identified in our study, which display opposing correlations between gene expression and DNA methylation.^[^ ^24^ ^]^ We selected two genes from each cluster and tested their upstream CRMs, which are characterized by DNA methylation.^[^ ^24^ ^]^ The results confirmed that the dCm system faithfully recapitulated the respective positive and negative correlations between DNA methylation and gene expression observed for these genes (Figure S13, Supporting Information). Together, these findings demonstrate the versatility of the dCm system in modulating CRMs from diverse biological sources via targeted DNA methylation, and highlight its broader potential to elucidate how DNA methylation shapes CRM‐mediated gene regulation.

### 2.4 Synthetic Reprogramming of Endogenous Yeast Genes through Methylation‐Dependent Logic Control

To evaluate whether the dCm system is capable of modulating endogenous gene expression in yeast, we selected *Suppressor of Sec One 1* (*SSO1*) and *SSO2*, whose mutations lead to temperature‐sensitive growth.^[^ ^75^ ^]^ Targeting these genes with the dCm system and their respective gRNAs resulted in pronounced growth retardation at elevated temperature (37 °C), as revealed by spot assays, whereas control groups transformed with empty vectors or maintained at 28 °C exhibited normal growth (Figure S14, Supporting Information). To further expand the applicability of the dCm system, we repaired the *ura3‐1* and *ade2‐1* mutations at their native loci in both W303a and W303α backgrounds using the WT alleles (Figures S15a, S16a, and S17a, Supporting Information). When these repaired loci were targeted by the dCm system using locus‐specific gRNAs, no discernible phenotypic changes were observed (data not shown), likely due to the inherent insensitivity of their native promoters to DNA methylation.

**Figure 5:**
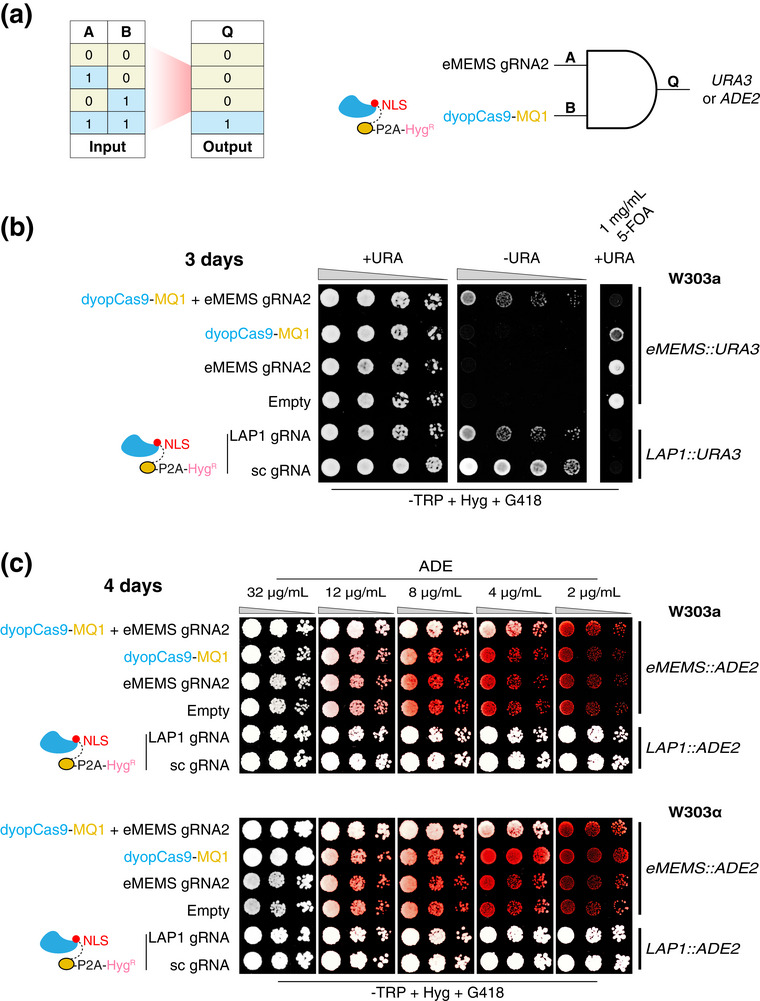
Synthetic Logic Gate Control of Endogenous Yeast Genes via dCm‐mediated DNA Methylation. a) Schematic of a two‐input AND gate. Left: Truth table showing that output Q is ON only when both inputs A and B are present. Right: Schematic implementation using the dCm system, where eMEMS gRNA2 (A) and dyopCas9‐MQ1 (B) converge at the *eMEMS::URA3* or *eMEMS::ADE2* locus to drive output Q (*URA3* or *ADE2* expression). b) Logic gate control of *URA3* expression by the dCm system configured as an AND gate. Strains harboring *eMEMS::URA3* were incubated under URA‐deficient conditions (‐URA) across four input combinations for 3 days. Conditional activation was further assessed by 5‐FOA counterselection in URA‐supplemented media. c) Logic gate control of *eMEMS::ADE2*. Strains were cultured across a gradient of ADE concentrations in both W303a (top) and W303α (bottom) backgrounds under four input combinations for 4 days. White colony pigmentation served as a metabolic readout of dCm‐mediated *ADE2* activation. Data represent tenfold serial dilutions from single colonies, with consistent outcomes observed in at least six out of eight colonies for each of the two independent experiments. *LAP1::URA3* and *LAP1::ADE2* strains served as methylation‐independent controls.

This prompted us to investigate whether synthetic CRMs could render these otherwise unresponsive endogenous loci regulatable by dCm‐mediated DNA methylation. We inserted eMEMS, ROM1, and LAP1 modules immediately upstream of the repaired *URA3* and *ADE2* coding regions, generating CRM‐driven expression strains (Figures S15a, S16a, and S17a, Supporting Information). Functional validation showed that insertion of eMEMS or ROM1 disrupted native promoter activity, while LAP1 supported constitutive expression, retaining the *URA3^+^* or *ADE2^+^* phenotype (Figures S15b, S16b, and S17b, Supporting Information). Using these engineered strains, we next tested whether the dCm system could implement Boolean logic control over endogenous loci. Our modular assembly framework enables the all‐in‐one plasmid construction of logic gates, such as a two‐input AND gate, by selectively substituting dCm components with inert adaptors to configure distinct input combinations (Figure S2a, Supporting Information). In the *eMEMS::URA3* background, yeast growth in URA‐deficient media was observed only when both dyopCas9‐MQ1 and eMEMS‐targeting gRNA2 were present, constituting the complete dCm system and consistent with AND gate behavior (**Figure** **5**a,b). This conditional activation was abolished by 5‐fluoroorotic acid (5‐FOA), confirming that *URA3* expression was driven by dCm‐mediated eMEMS activation (Figure 5b). Similarly, at the *eMEMS::ADE2* locus, dual‐input cells formed white colonies at ADE concentrations as low as 4  µg mL^−1^ across both strain backgrounds within 3–4 days, indicating effective dCm‐mediated activation (Figure 5a,c; Figure S18, Supporting Information). By contrast, strains with only one or no input exhibited pronounced metabolic defects even at 12  µg mL^−1^ ADE (Figure 5 a,c; Figure S18, Supporting Information). As expected, strains bearing LAP1 upstream of these loci were unresponsive to dCm inputs, consistent with its methylation‐independent activation mode (Figure 5a,c; Figure S18, Supporting Information). Together, these results establish a generalizable framework for synthetic epigenetic control of both native and heterologous genes in yeast, enabling precise and programmable gene regulation via engineered DNA methylation logic.

## 3 Discussion

In this study, we established modular frameworks for targeted epigenome editing of CRMs, enabling their functional interrogation both in situ and within synthetic configurations that reveal regulatory portability across species. Our toolkits demonstrated robust efficacy in editing diverse CRMs, ranging from well‐characterized but previously unedited elements to uncharacterized ones, via targeted DNA methylation and demethylation. Using the yeast platform lacking native DNA methylation, we also showcased controllable epigenome editing, uncovered epigenetic crosstalks influencing gene expression, and achieved logic‐based, DNA methylation‐dependent control of endogenous gene expression.

### 3.1 Epigenome Editing via DNA Demethylation and Methylation in Planta

We evaluated the efficacy of our dCd system in epigenome editing of biologically impactful CRMs (Figure 3; Figures S1 and S2, Supporting Information), including the epigenetic regulation region of the CaMV *35S* promoter,^[^ ^16^, ^17^, ^18^, ^35^ ^]^ the heterochromatin region of the long intron of *IBM1*,^[^ ^17^, ^33^, ^34^, ^35^ ^]^ and the sensor region of the *A. thaliana* DNA methylstat.^[^ ^18^, ^30^ ^]^ These regions exemplify the functional diversity of DNA methylation‐responsive CRMs, spanning activation, repression, and alternative splicing modulation. Our results showed that the dCd system effectively erased DNA methylation in the distal region of the CaMV *35S* promoter to promote *SUC2* transgene expression (Figure 3b,c; Figure S3c, Supporting Information), reduced functional IBM1 isoform levels to postpone bolting time (Figure 3d), and blocked transcriptional activation driven by eMEMS transplanted into a synthetic interspecies context (Figure 3e–h; Figure S4, Supporting Information). These findings suggest that our DNA demethylation‐based epigenome editing strategy, empowered by the flexibility and programmability of reconfigurable framework (Figure 1; Figure S1, Supporting Information), is broadly applicable across diverse molecular scenarios, enabling modulation of regulatory networks and manipulation of targeted traits.

The human demethylase TET1 facilitates DNA demethylation by catalyzing the oxidation of 5‐methylcytosine (5mC) to its oxidized derivatives, which can lead to passive demethylation during replication or active removal via base excision repair.^[^ ^76^ ^]^ Its catalytic domain, TET1cd, has been employed in plants to direct locus‐specific demethylation at the promoter of *FLOWERING WAGENINGEN* (*FWA*) and the methylated region of the *CACTA1* transposon, using either zinc finger (ZF) fusions or dCas9‐SunTag systems.^[^ ^51^, ^52^ ^]^ TET1cd has also been tethered directly to dCas9 to form a dCas9‐TET1cd fusion capable of erasing epigenetic memory marks at remethylable loci.^[^ ^54^ ^]^ In our study, we further expanded the application of dCas9‐TET1cd fusion beyond previously tested canonical targets, applying it to a panel of CRMs with distinct chromatin contexts. The catalytic dependence was confirmed by the restoration of eMEMS‐driven reporter activation upon TET1cd mutation or deletion (Figure 3g,h; Figure S4c, Supporting Information), supporting its functional necessity in targeted epigenome editing via DNA demethylation.

In mammalian cells, the CRISPR/dCas9 system has been adapted to direct site‐specific DNA methylation.^[^ ^44^, ^45^, ^77^, ^78^ ^]^ Building on our modular framework, we attempted to implement targeted DNA methylation in planta using *A. thaliana* DRM2 and DRM2cd as methylation effectors (Figures S1 and S5, Supporting Information). However, these constructs did not alter the activity of the eMEMS‐coupled reporter in the *N. benthamiana* transient system (Figure S5, Supporting Information). Given the robust activity of eMEMS, observed even with sc gRNAs (Figure 3f–h; Figure S5, Supporting Information), it is plausible that the endogenous DNA methylation machinery in *N. benthamiana* may already be functionally engaged with the eMEMS module. This intrinsic engagement could saturate the capacity of eMEMS to respond to additional methyltransferase input, thereby masking any potential enhancement. Consequently, targeted delivery of DRM2 or DRM2cd failed to further elevate eMEMS activity.

It is noteworthy that in the dCas9‐SunTag system, the *Nicotiana tabacum* DRM methyltransferase catalytic domain (NtDRMcd) exhibited observable targeted methylation activity at the CRMs of the *FWA* and *SUPERMAN* loci.^[^ ^53^ ^]^ However, these outcomes were reported in specific genetic contexts, such as *fwa* epimutants or the *Landsberg erecta* (Ler) ecotype, where native DNA methylation is absent at the targeted regions.^[^ ^53^ ^]^ This highlights the baseline methylation state as a critical factor in determining the efficacy of targeted DNA methylation editing. Indeed, irrespective of the delivery modalities, whether using dCas9‐SunTag or ZF‐based platforms, most reported DNA methylation‐directed epigenome editing strategies, including co‐targeting of multiple DNA methylation effectors,^[^ ^59^ ^]^ use of the potent bacterial DNA methyltransferase MQ1^Q147L^,^[^ ^60^ ^]^ and systematic screening of silencing effectors,^[^ ^58^ ^]^ have been implemented primarily in *fwa* epialleles, where the absence of native DNA methylation minimizes interference and facilitates targeted modification.

### 3.2 DNA Methylation Editing and Methylation‐Driven Expression in Non‐DNA Methylated Contexts

Endogenous DNA methylation in planta appears to constrain the utility of CRISPR/dCas9‐mediated targeted DNA methylation for CRM validation. To overcome this limitation, we developed an alternative dCm framework tailored to the DNA methylation‐free model organism *S. cerevisiae*.^[^ ^63^, ^64^ ^]^ The constructed dCm system successfully activated chimeric eMEMS reporters across multiple yeast genotypes in a DNA methylation‐dependent manner, as evidenced by its sensitivity to DNA methylation inhibitors (Figure 4a,b; Figures S6–S8, Supporting Information), faithfully recapitulating the regulatory function of methylated eMEMS as originally observed in *A. thaliana*.^[^ ^18^, ^30^ ^]^ Functional reconstitution in heterogeneous systems was further validated using orthologous eMEMS from a different ecotype and species (Figure S9, Supporting Information), suggesting a conserved mode of eMEMS action and affirming the utility of the dCm platform. In addition, the dCm system was tunable through optogenetic and temperature‐sensitive control using anti‐CRISPR‐specific inhibitors (Figure 4c–e; Figures S10 and S11, Supporting Information), demonstrating its versatility for CRM validation and for constructing adaptable and reversible eMEMS‐based synthetic circuits. Furthermore, the platform enabled mechanistic interrogation of epigenetic interactions governing gene expression (Figure 4g; Figure S12, Supporting Information). Its robustness was further supported by successful applications to CRMs derived from crop and viral sources, as well as yeast endogenous genes (Figure 4h; Figures S13 and S14, Supporting Information), demonstrating its broad applicability for characterizing CRMs of diverse origins.

Delineating the causal impact of epigenetic modifications in CRMs is essential for dissecting gene regulatory mechanisms. Utilizing our dCm system, we identified a causal relationship between reporter activation and DNA methylation at the CRMs of certain genes from two tomato gene clusters (Figure S13, Supporting Information).^[^ ^24^ ^]^ Although omics data suggested correlative patterns between DNA methylation and gene expression in the two clusters,^[^ ^24^ ^]^ a significant number of genes did not exhibit reporter responses to DNA methylation (data not shown), suggesting that DNA methylation at their CRMs may be insufficient or non‐causal for gene regulation. These findings underscore the importance of experimental validation when assigning function to epigenetic marks and highlight the utility of the dCm system as a causal interrogation platform. Similar to recently developed modular epigenome editing platforms for identifying functional histone modifications in mammalian systems,^[^ ^48^ ^]^ our approach enables the dissection of DNA methylation contributions to CRM‐mediated transcriptional regulation in a controlled, context‐resolved manner.

The eMEMS DNA methylation‐driven expression likely represents an evolutionary adaptation essential for maintaining epigenetic homeostasis.^[^ ^31^, ^32^ ^]^ While yeast lacks endogenous DNA methyltransferases due to purifying selection during evolution, the introduction of mammalian enzymes has been shown to establish DNA methylation patterns akin to those in mammals,^[^ ^79^, ^80^, ^81^ ^]^ thereby modulating gene expression in yeast.^[^ ^63^, ^81^, ^82^, ^83^ ^]^ Moreover, genes involved in meiosis and nonsexual flocculation in S. cerevisiae display a positive correlation between their expression levels and DNA methylation,^[^ ^63^, ^82^, ^83^ ^]^ suggesting the presence of an as‐yet‐uncharacterized DNA methylation recognition mechanism, either evolved independently of or selected over DNA methyltransferases. This intrinsic recognition capacity may underlie the responsiveness of multiple methylated eMEMS and tomato‐derived CRMs in yeast (Figures 4 and 5; Figures S6–S9 and S13a, Supporting Information). Elucidating this recognition mechanism could deepen our understanding of yeast's epigenetic adaptability and open new avenues for engineering eMEMS‐based platforms in synthetic biology.

### 3.3 DNA Methylation as a Regulatory Layer in Synthetic Circuit Design

For decades, S. cerevisiae has served as a foundational chassis for the development of synthetic genetic circuits, enabling high‐titer production of biofuels and therapeutic compounds.^[^ ^84^, ^85^ ^]^ The recent advent of gene editing technologies, especially CRISPR/Cas systems, has propelled the construction of programmable genetic circuits in yeast, offering precise control over gene expression.^[^ ^86^, ^87^, ^88^, ^89^ ^]^ Leveraging the modularity of CRISPR/dCas9, NOR gates have been developed in yeast through combination with the transcriptional repressor Mxi1, enabling multilayered cascade regulation with minimal transcriptional leakage in the OFF state due to the specificity of gRNA inputs.^[^ ^89^ ^]^ In parallel, a tightly regulated AND gate has been implemented by combining a β‐estradiol‐inducible MCP‐VP64 fusion, a galactose‐inducible dCas9, and gRNAs containing MS2 RNA aptamers.^[^ ^88^ ^]^ This system enables fine‐tuned expression control, contingent upon the simultaneous presence of both chemical inducers.^[^ ^88^ ^]^ Similarly, a multilayered cell‐cell communication circuit has been established in yeast using auxin degron‐mediated regulation. In this system, dCas9‐Mxi1 and dCas9‐VP64 were fused to auxin‐responsive degrons to serve as tunable repressor and activator modules, respectively, enabling precise modulation of gene expression in response to auxin.^[^ ^87^, ^90^ ^]^

While existing synthetic circuit designs have achieved remarkable precision and programmability, they primarily operate through transcriptional effectors and lack integration with epigenetic states, particularly DNA methylation, as a regulatory dimension. In contrast, we incorporated DNA methylation into circuit design, exemplified by a constitutive variant of the dCm‐based AND gate that modulates endogenous gene expression without detectable leakage (Figure 5; Figure S18, Supporting Information). This inspires a new paradigm in which dynamic epigenetic regulation serves as a programmable layer within engineered circuits, broadening the design space of synthetic biology. While our AND gate configuration lacks signal‐responsiveness, it establishes the feasibility of using DNA methylation to encode stable logical states. This constitutive mode of control could serve as a noise‐free, signal‐independent bio‐circuit primitive, suitable for benchmarking or for embedding into hierarchical synthetic networks where stability is prioritized over dynamic responsiveness. At the same time, our versatile framework readily supports the incorporation of inducible components for constructing signal‐responsive circuits (Figure 1; Figure S1, Supporting Information).

Moreover, the optogenetic and temperature‐sensitive modulation is readily accommodated by our dCm system, endowing genetic circuits with tunability (Figure 4c–e; Figures S10 and S11, Supporting Information). The autonomous functionality of eMEMS, conferred by dCm‐mediated targeted methylation, effectively bypasses reliance on traditional transcriptional regulators,^[^ ^87^, ^88^, ^90^ ^]^ thereby enhancing circuit stability and regulatory specificity. Furthermore, the dual‐faceted nature of eMEMS in mediating both transcriptional activation and repression through distinct DNA methylation states offers a unique mechanism for constructing self‐regulating feedback loops (Figures 3, 4, 5). These loops could dynamically and reversibly adjust transcriptional load and enhance cellular sustainability, enabling the design of industrial microbial cell factories with self‐sustained resilience. Finally, the absence of native DNA methylation ensures minimal interference between native cellular processes and dCm‐mediated, eMEMS‐coupled circuits, conferring orthogonality for the precise modulation of synthetic signaling networks. Collectively, our approach augments the functional repertoire of synthetic gene circuits, with the potential to open avenues for adaptable applications in biotechnology and biomedicine.

In conclusion, our dCd and dCm systems efficiently manipulate diverse CRMs both in planta and in yeast, demonstrating their capability to rewrite regulatory elements and tailor specific traits. Looking ahead, integrating a broader range of epigenetic effectors, such as histone modifiers, alongside novel Cas protein variants with our modular frameworks holds great promise for advancing the realm of epigenome editing. Deploying these advanced systems across diverse biological contexts may deepen our understanding of the intricate and dynamic interconnections between the epigenome and *cis*‐regulome, while translating this knowledge into innovative solutions for pressing challenges in applied fields.

## 4 Experimental Section

### Plant and Yeast Materials

A previously established transgenic plant harboring *35S::SUC2* was utilized.^[^ ^17^, ^18^ ^]^ Mutants *ros1‐4* and *ibm1‐4* were obtained from ABRC. The *ros1‐4* mutant was crossed with *35S::SUC2*, and homozygous alleles were identified. Transgenic lines at the T1 generation were screened on half‐strength Murashige and Skoog (MS) medium plates supplemented with 1% (wt/vol) glucose or sucrose, 1% (wt/vol) agar, and 7.5 µg mL^−1^ glufosinate ammonium (Sigma, 45520‐100MG). Plates were incubated in a growth chamber at 22 °C under 100 µmol⋅m^−2^⋅s^−1^ fluorescent light (16 h light/8 h dark) for 10 days following 2 days of stratification at 4 °C. Materials transformed with constructs of dCd‐*35S* gRNAs and dCd‐*IBM1* gRNAs (Figure 3; Figure S3, Supporting Information) were transferred from plates to soil in a growth room under conditions similar to those in the growth chamber, and bolting time was analyzed. Transgenic lines carrying dCd‐*35S* gRNAs at the T2 generation, cultured on plates, were subjected to bisulfite sequencing and real‐time PCR analysis after 10 days of growth. All plant materials or CRMs were derived from the Col‐0 background, unless otherwise stated.

Yeast strains Y1H Gold (Takara) and W303a/α (Dharmacon, Horizon Discovery) were used for yeast‐based experiments. Mutants of W303a/α were generated or further re‐engineered with CRMs via homologous recombination with amplified fragments containing a G418 selection marker (ThermoFisher), using the specified primer pairs listed in Table S1, Supporting Information.

### Framework and Vector Construction for Plants

DNA oligos were synthesized for specific experimental purposes, as detailed in Table S1, Supporting Information. PCR amplification of construct fragments was conducted using PrimeSTAR GXL DNA Polymerase (Takara). The frameworks for constructing CRISPR/Cas‐based targeted epigenomic editing of CRMs are illustrated in Figures 1 and 2, with additional details provided in Figure S1 and S2 as well as Tables S1 and S2, Supporting Information. Briefly, using In‐Fusion Snap assembly technology (Takara), a dual‐selection destination vector, pGW303‐mPAT, was generated by inserting a *sacB‐gentamycin* resistance (GmR) cassette, flanked by *Aar*I recognition sites, into the *Sbf* I site of the original pGW303 vector to enable Golden Gate assembly. The *Aar*I site within the *PHOSPHINOTHRICIN ACETYLTRANSFERASE* (*PAT*) gene was eliminated (indicated by a solid circle; Figure S1, Supporting Information). Components of the CRISPR/Cas *Epi*‐effector module, designed with sequential *Aar*I sites, were first cloned into the pJET1.2 vector (ThermoFisher). Mutations in Cas9 and TET1cd were introduced using the QuikChange Site‐Directed Mutagenesis method (Agilent). These intermediate constructs were sequentially assembled into pGW303‐mPAT using Golden Gate technology through the *sacB*‐*GmR* cassette (Figure S1; Table S2, Supporting Information). An example sequence for the *AtUBQ1* promoter‐driven dCas9‐TET1cd fusion is provided in Note S1, Supporting Information. Four gRNA donor cassettes containing back‐to‐back *Bsa*I sites upstream of an optimized gRNA scaffold,^[^ ^91^ ^]^ along with corresponding adaptors, were synthesized and cloned into the pLZ‐*sacB* vector via *Sap*I sites (Figure S1; Table S2 and Note S2, Supporting Information). Specific gRNA oligos (Table S1 and Note S2, Supporting Information) were annealed and inserted into these gRNA donor vectors via *Bsa*I sites. The resulting gRNA donor vectors, or adaptors if needed, can be incorporated into the dCd or dCm constructs through Gateway technology (ThermoFisher). Alternatively, these gRNA donor vectors or adaptors can be cloned into the custom intermediate vector pLZENTR‐AARI using Golden Gate technology, either with or without the CRM reporting system (Figure S1, Supporting Information). In the CRM reporting module, CRMs can be positioned upstream of reporter genes, such as Luc. All blocks used for reporting can be cloned into the pJET1.2 vector, among which a space‐filled linker allows the construction of dual reporter systems (Figure S1; Tables S1 and S2, Supporting Information). An example sequence assembling the eMEMS‐*35S* mini pro‐Luc and pLZ‐Donor II is provided in Note S3, Supporting Information. The pLZENTR‐AARI intermediate vectors containing both gRNA and reporting modules were assembled into the dCd or dCm system via Gateway technology (Figure S1, Supporting Information). The final constructs were transformed into *A. thaliana* or *N. benthamiana* via routine stable or transient transformation protocols.

### Framework and Vector Construction for Yeast

The construction schemas of CRISPR/dCas‐based toolkit for targeted epigenomic editing of CRMs in yeast are analogous to those used in planta (Figure 2; Figure S2, Supporting Information), and relevant sequences are provided in the Notes S4 and S5, Supporting Information. Specifically, four independent blocks were designed and cloned into the intermediate pJET1.2 vector, each representing an essential component of the CRISPR/dCas *Epi*‐effector module (Figure S2a, Supporting Information). To facilitate the enrichment of yeast cells expressing the CRISPR/dCas *Epi*‐effector module, a P2A auto‐cleavage peptide was employed to introduce FPs or antibiotic resistance genes. In the gRNA module, three gRNA expression cassettes and their adaptors were developed, each driven by the *SNR52* RNA polymerase III (Pol III) promoter and flanked by inverted *Bsa*I sites for annealed gRNA oligo insertion (Figure S2a, Table S2 and Note S5, Supporting Information). *Epi*‐effector cassettes were integrated into pAG434GAP‐ccdB‐HA (Addgene) for strong expression or into the modified pLZ‐434ROX3‐ccdB‐HA for moderate expression, along with the specified gRNA cassettes via the pLZENTR‐*AAR*I intermediate vector (Figure S2a; Table S2, Supporting Information). An example sequence of the dCm system with pLZ‐Y‐Donor II delivering eMEMS gRNA2 (dyopCas9‐MQ1‐Hgy^R^‐eMEMS gRNA2) is provided in Note S4, Supporting Information. CRMs were amplified from the genomes of *A. thaliana* and the tomato cultivar Micro‐Tom or from plasmids, and integrated into the pLZ‐AbAi‐GW, a Gateway system previously modified in our lab. This vector uses a sensitive antibiotic resistance gene (*AUR‐1C*) as a reporter (Table S2, Supporting Information). In this study, it was further engineered to generate the pLZ‐DF‐GW using specific restriction sites (Figure S2; Tables S1 and S2, Supporting Information). Anti‐CRISPR inhibitors were amplified from designated plasmids and expressed using a custom‐designed pLZ14252‐KanMx vector (Tables S1 and S2, Supporting Information). All plasmids were propagated in TOP10 or One Shot *ccdB* Survival 2 T1^R^ strains (ThermoFisher), manipulated using the Presto Mini Plasmid Kit (Gene Aid), and verified by Sanger sequencing at the Purdue Genomics Core Facility.

### Yeast Assays

CRM reporters were integrated into the yeast genome following linearization. Positive transformants were identified and subsequently transformed with the dCm system, with selection performed on SD‐URA‐TRP medium supplemented with 200 µg mL^−1^ Hygromycin B (Hyg; H3274‐1G, Sigma). The effects of the dCm system on CRMs were evaluated on the same medium containing AbA (Takara) at the specified concentrations, which are known to be toxic to yeast even at low levels. Selection for anti‐CRISPR inhibitor expression was performed on plates containing 400 µg mL^−1^ G418 (ThermoFisher). To assess DNA methylation dependence, three DNA methylation inhibitors were used: 5‐Aza‐2′‐deoxycytidine (5‐AzadC; Sigma, A3656‐10MG), 5‐Azacytidine (5‐Aza; Sigma, A2385‐100MG), and Zebularine (Sigma, Z4775‐25MG). Extensive screening of *A. thaliana* genomic fragments was conducted to establish reliable experimental controls. Fragments that failed to support yeast growth at low AbA concentrations (50 ng mL^−1^) were designated as optimal negative controls (e.g., a 209‐nt fragment from *RHOMBOID‐RELATED INTRAMEMBRANE SERINE PROTEASE 1, ROM1*), while fragments that supported growth at high AbA concentrations (500 ng mL^−1^) were designated as optimal positive controls (e.g., a 268‐nt fragment from *LAP1*). The positive controls functioned by activating the AbA‐resistant (*AbA^R^*) gene (*AUR1‐C*) on the pLZ‐AbAi‐GW reporting vector. For each group, at least eight independent colonies were randomly selected and suspended in sterile water. The resulting suspensions were plated onto selection and reporting media containing AbA. Colonies exhibiting consistent growth or no growth in the presence of AbA (at least six out of eight) were considered indicative of active or inactive *AbA^R^* reporter expression, respectively. Each experiment was performed with a minimum of two biological replicates. All assays were validated in at least two independent yeast strains, Y1H Gold (Takara) and W303a/α (Dharmacon, Horizon Discovery), following stringent experimental standards. One representative colony from each group was selected for imaging under either gradient dilutions or an undiluted sample, as indicated. Unless otherwise stated, all yeast cultures were grown at 28 °C in darkness.

### Analyses of the Efficiency of Epigenome Editing by dCd and dCm Systems

The demethylation efficiency of the dCd system was analyzed using high‐throughput amplicon sequencing (“Wide‐seq”) at the Purdue Genomics Core Facility. Genomic DNA was extracted from transgenic lines carrying dCd‐*35S* gRNAs and corresponding control lines, followed by bisulfite conversion using the EpiTect Bisulfite Kit (QIAGEN). The converted DNA was subjected to nested PCR amplification with primers listed in Table S1, Supporting Information. Amplified products were purified and analyzed using Wide‐seq. Methylated cytosines were classified into CG and non‐CG contexts, and processed using Bismark. To assess the effect of dCd on *SUC2* expression, total RNA was extracted and subjected to RT‐PCR and real‐time RT‐PCR using primers detailed in Table S1, Supporting Information, as previously described.^[^ ^18^ ^]^ For transient expression assays in *N. benthamiana*, chemiluminescence imaging of luciferin‐sprayed leaves was performed using a CCD camera (Princeton Instruments) to evaluate the activity of both dCd and dCm systems. Protein expression levels of anti‐CRISPR inhibitors were measured in yeast grown under specified conditions. Protein extraction was performed using the glass bead method. Extracts were denatured by boiling for 10 min in 1 × SDS loading buffer, separated by SDS‐PAGE, and subjected to immunoblotting with anti‐HA and anti‐GAPDH antibodies (Abcam).

### Statistical Analysis

Unless otherwise stated, all data are presented as mean ± SD. Comparisons between two groups were performed using a two‐tailed unpaired Student's *t‐*test. Statistical significance was defined as *p* < 0.01 or *p* < 0.001. All analyses were conducted using GraphPad Prism 8.

## Conflict of Interest

The authors declare no conflict of interest.

## Author Contributions

L.Z. and J.‐K.Z. conceived and designed research; L.Z., J.F., T.L., C.Z., F.F., and Z.L. performed research; L.Z. and J.‐K.Z. analyzed data and wrote the paper. All authors read and approved the manuscript.

## Supporting information



Supporting Information

## Data Availability

The data that support the findings of this study are available in the supplementary material of this article.
