# Supplementary material for: A Modular and Customizable CRISPR/Cas Toolkit for Epigenome Editing of Cis‐regulatory Modules
Source: Adv Sci (Weinh). 2025 Sep 29;12(47):e03917. doi: 10.1002/advs.202503917 (PMC12713107; doi:10.1002/advs.202503917)
Supplement: Supplementary file 1 — Supporting Information [file ADVS-12-e03917-s001.docx]

**Supporting Information for:**

**A Modular and Customizable CRISPR/Cas Toolkit for Epigenome Editing of *cis*-regulatory Modules**

*Lingrui Zhang, Jianxin Fu, Tiandan Long, Chao Zhang, Fuhua Fan, Zhaobo Lang, and Jian-Kang Zhu**

L. Zhang

Department of Horticulture and Landscape Architecture, Purdue University, West Lafayette, IN 47907, USA (at the time of research); ORCID: 0009-0004-3007-2811

Z. Lang, J.-K. Zhu

Institute of Advanced Biotechnology, Institute of Homeostatic Medicine, and School of Medicine, Southern University of Science and Technology, Shenzhen 518055, PR China

Email: [zhujk@sustech.edu.cn](mailto:zhujk@sustech.edu.cn); ORCID: 0000-0001-5134-731X

J. Fu, C. Zhang

College of Landscape Architecture, Zhejiang Agriculture and Forestry University, Hangzhou 311300, PR China

T. Long

State Key Laboratory of Crop Gene Exploration and Utilization in Southwest China, Sichuan Agricultural University, Chengdu 625014, PR China

F. Fan

Institute for Forest Resources and Environment of Guizhou, Guizhou University, Guiyang 550025, PR China

**This File Includes the Cover Page and:**

Figures S1 to S18

Tables S1 and S2

Supplemental Sequence Notes 1 to 5

SI References

**Figure S1. Framework for Customizable Targeted Epigenome Editing of CRMs in Planta.** Step I (grey arrowhead): Modifying pGW303 to generate the dual-selection destination vector, pGW303-mPAT. Step II (orange arrowheads): Assembly of the CRISPR/dCas *Epi*-effector module by individually cloning five essential blocks into an intermediate vector, followed by ordered insertion into pGW303-mPAT via *Aar*I sites. Cas proteins were split into N-terminal (N-Cas) and C-terminal (C-Cas) fragments to facilitate the introduction of mutations. Step IIIa (black arrowheads): Direct integration of up to four gRNA cassettes from the pLZ-Donor vectors into pGW303-mPAT via Gateway technology. Adaptors can replace donor vectors to vary the number of expressed gRNAs. Step IIIb (blue arrowheads): Integration of gRNA donor vectors or adaptors into the custom intermediate vector pLZENTR-AARI via Golden Gate assembly, followed by transfer into pGW303-mPAT using Gateway technology. Step IIIc (red dashed arrowheads): Integration of gRNA donor cassettes and CRM reporting blocks into pLZENTR-AARI prior to final transfer. Reporter genes, such as luciferase (Luc), were designed either with or without *35S* minimal promoter (*35S* mini pro; green shape). Landmarks of 4 nt at fragment ends or specified positions define *Aar*I-mediated Golden Gate assembly sites. Detailed sequences and additional information are available in Experimental Section and Supplemental Sequence Notes 1-3, Tables S1 and S2 (Supporting Information).

**Figure S2. Framework of Customizable Systems for Epigenome Editing of CRMs in *S. cerevisiae*. (a)** Four modular blocks constitute CRISPR/dCas *Epi*-effector module used in yeast. FPs or antibiotic resistance genes, linked via a P2A auto-cleavage peptide, enrich properly expressed cells. Three gRNA expression cassettes, driven by the *SNR52* Pol III promoter, and corresponding adaptors are sequentially inserted into the entry vector pLZENTR-AARI via *Aar*I sites, along with the dCas *Epi*-effector system. The entry vector is compatible with Gateway expression systems in yeast, such as pAG434GAP-ccdB-HA with a strong *GAP* promoter, and pAG434ROX3-ccdB-HA modified to include a milder *ROX3* promoter to accommodate diverse expression requirements. **(b)** The CRMs of interest are integrated into the pLZ-AbAi-GW or a newly-developed pLZ-DF-GW. The reporter systems are introduced into the yeast genome through homologous recombination (HR) between the *URA3* sequence and the engineered *ura3-1* locus in yeast strain following vector linearization. Detailed sequences and additional information are available in Experimental Section and Supplemental Sequence Notes 4-5, Tables S1 and S2 (Supporting Information).

**Figure S3. Efficiency of the dCd System in DNA Demethylation and Gene Expression Regulation, and Analysis of DNA Methylation Pattern and gRNA Targeting Sites in *IBM1* Intron. (a)** Diagram of the transgene expressing *SUC2* under the *35S* promoter (*35S* pro) in *A. thaliana*. gRNA targeting sites are denoted by short magenta lines. Regions A and B are defined based on DNA methylation profiles.^[1, 2]^ **(b)** DNA methylation levels at the *35S* promoter Region B in the indicated genotypes were analyzed by Wide-seq of PCR amplicons from bisulfite-converted genomes. The *ros1-4* mutant is in the *35S::SUC2* background. **(c)** Expression of *SUC2* in the indicated genotypes. Values represent means ± SD from three biological replicates, normalized to *UBQ5* and expressed as fold-change relative to *35S::SUC2* controls (set to 1) across genotypes and analyzed using an unpaired two-tailed Student’s test. **(d)** IGB snapshot showing the DNA methylation pattern in *IBM1*. The genomic structure of *IBM1* is depicted below, highlighting transcript variation arising from alternative distal and proximal polyadenylation. gRNAs targeting sites within the heterochromatin intron are indicated by short blue lines.

**Figure S4. Specific Modulation of eMEMS-coupled Reporter by the dCd System in Planta. (a)** Additional replicates demonstrate that the dCd system suppressed the eMEMS-*35S* mini-Luc reporter with eMEMS gRNA2, whereas this suppression was blocked by the TET1cd mutation **(c)**. **(b, d)** In contrast to eMEMS-*35S* mini-Luc reporter **(b)**, the dCd system failed to suppress the LAP1-*35S* mini-Luc reporter with LAP1 gRNA **(d)**. Black arrowheads indicate the expression orientation of gRNA delivery cassettes, while blue arrowheads denote the orientation of CRM reporting modules, represented by empty rectangles. CRM luciferase reporter modules are sketched independently of dCd and gRNA cassettes, positioned on the left or right side of the luminescent images. Short lines above the *cis*-elements mark the gRNA targeting sites.

**Figure S5. Testing the dCm System for Manipulating eMEMS-Equipped Reporter in Planta. (a, b)** DRM2 **(a)** and DRM2cd **(b)** replaced TET1cd in the dCd system to generate dCm constructs for use in planta. Target gRNA2 and three sc gRNAs were used to test the effect on the eMEMS-*35S* mini-Luc reporter. Empty rectangles denote the CRM luciferase reporter and gRNA cassettes, which are sketched independently of dCm on the left of the luminescent images. Short lines above the *cis*-elements indicate gRNA targeting sites.

**Figure S6. The dCm System Activates eMEMS-coupled Reporter in a DNA Methylation-Dependent Manner, Substantiated by 5-AzadC Treatment in Three Yeast Strains.** The eMEMS exhibited activity only when both the dCm and target gRNA2 were present, and this activation was inhibited in a dose-dependent manner by 5-AzadC. No activation was observed with sc gRNAs. These findings were obtained across different yeast genotypes. In the LAP1 control groups, no influence on reporter activation was observed regardless of gRNAs, genotypes, or chemical treatment. Each data point represents a single colony, with consistent outcomes observed from at least six out of eight independent colonies per experiment. Each experiment was repeated at least twice. Colonies outlined by magenta dashed boxes are shown in Figure 4a.

**Figure S7. The dCm System Activates eMEMS-coupled Reporter in a DNA Methylation-Dependent Manner, Substantiated by 5-Aza Treatment in Three Yeast Strains.** Legend details are identical to Figure S6, except 5-Aza was used as the DNA methylation inhibitor in this experiment.

**Figure S8. Zebularine Treatment Confirms the DNA Methylation-dependent Activation of eMEMS by the dCm System in Three Yeast Strains.** Legend details are identical to Figures S6 and S7, except zebularine was used as the DNA methylation inhibitor in this experiment.

**Figure S9. The dCm System Enables Epigenome Editing of eMEMS Orthologs.** Orthologous eMEMS sequences were derived from Col-0, C24, and *A. lyrata*. *AbA^R^* reporter activity was assessed in the Y1H Gold strain under varying concentrations of AbA in the presence of the respective target gRNA or sc gRNA. LAP1 and ROM reporters served as positive and negative controls, respectively.

**Figure S10. Inhibition of eMEMS-coupled Reporter Activation by Specific Anti-*S. pyogenes* Cas9 Inhibitors in the W303a Strain.** Activation of the eMEMS reporter with target gRNA2 was sensitive to specific anti-*S. pyogenes* Cas9 Inhibitors but not to non-specific inhibitors in the W303a strain.^[3, 4]^ Yeast cultures grown at 28^o^C in darkness ensured AcrIIA4-LOV2 activity,^[5]^ while AcrIIA2 remained unstable,^[4]^ resulting in weak suppression of eMEMS activity. The LAP1 control group showed no effect from inhibitors. Each data point represents four gradient dilutions of a single colony, with consistent outcomes from at least six of eight colonies per experiment, conducted at least twice. The initial gradients boxed are shown in Figure 4c.

**Figure S11. Inhibition of eMEMS-coupled Reporter Activation by Specific Anti-*S. pyogenes* Cas9 Inhibitors in the W303α** **Strain. (a)** Similar to W303a, eMEMS activation by the dCm system with target gRNA2 was sensitive to specific inhibitors in the W303α strain.^[3, 4]^ For detailed descriptions, refer to the legend of Figure S10. **(b)** AcrIIA4-LOA inhibition was nullified under light conditions, while **(c)** AcrIIA2 inhibition was enhanced at 22°C. The LAP1 control group showed no effect under any conditions. Each data represents three gradient dilutions of a single colony, with consistent outcomes from at least six of eight colonies per experiment, conducted at least twice.

**Figure S12. Set2 is essential for the eMEMS-coupled Reporter Activation by the dCm System.** The dCm system alongside eMEMS gRNA2 failed to activate the eMEMS reporter in *set2Δ* mutant of W303α while activation was unaffected in *set1Δ* and *swd1Δ* mutants in W303a/**α**. Each data point represents four gradient dilutions of a single colony, with consistent outcomes from at least six of eight colonies per experiment, conducted at least twice.

**Figure S13. Generality of the dCm System in Epigenome Editing of CRMs. (a)** Two genes from tomato cluster II, based on *k*-means clustering, which show higher expression with DNA methylation,^[6]^ were tested using their CRMs. **(b)** Two cluster I genes, suppressed by increased DNA methylation, were similarly tested.^[6]^ Their CRMs and eMEMS control were tested in triplicates, with primers listed in Table S1 (Supporting Information). Each data point represents four gradient dilutions of a single colony, with consistent outcomes from at least six of eight colonies per experiment, conducted at least twice.

**Figure S14. dCm-Mediated Epigenome Editing of Endogenous *SSO1* and *SSO2* in Yeast.** The dCm system was targeted to endogenous *SSO1* and *SSO2* loci in W303a (top) and W303α (bottom) backgrounds using specific gRNAs. Growth retardation was assessed at 37°C (right) relative to 28°C (left). Strains transformed with empty vectors served as positive controls. Each data point represents a tenfold serial dilution from a single colony, with consistent outcomes from at least six of eight colonies per experiment, conducted at least twice.

**Figure S15. Repair of *ura3-1* and Construction of CRM-driven *URA3* Strain in W303a. (a)** Schematic of a two-step genome engineering strategy. Step I: The *ura3-1* allele in W303a was repaired by HR with the WT *URA3* allele to restore *URA3⁺.* Step II: Synthetic CRM modules, together with a KanMX6 expression cassette for selection, were inserted between the promoter and coding region of the repaired *URA3* locus via HR. The genomic location of *URA3* is indicated. Resulting genotypes are shown. Primers used for PCR screening and sequencing are indicated, with corresponding sequences provided in Table S1 (Supporting Information). **(b)** Functional validation of the constructed strains. Left: Growth on G418-containing media confirmed correct integration in engineered strains after 2 days at 28°C. Right: Growth on URA-deficient or URA- and 5-FOA-supplemented media assessed *URA3* activity across the indicated genotypes after 3 days. Each data point represents a tenfold serial dilution from a single colony. Successfully validated strains were archived at -80°C for downstream applications.

**Figure S16. Repair of *ade2-1* and Construction of CRM-driven *ADE2* Strain in W303a. (a)** The same two-step genome engineering strategy described in Figure S15a (Supporting Information) was used to repair the *ade2-1* mutation and insert synthetic CRM modules upstream of the restored *ADE2* locus in W303a. The genomic location of *ADE2* is indicated. **(b)** Functional validation of the constructed strains. Middle: Growth on G418-containing media confirmed correct integration after 2 days at 28°C. Bottom: Colony color across an ADE concentration gradient reflected *ADE2* activity in the indicated genotypes after 3 days. Each data point represents a tenfold serial dilution from a single colony. Successfully validated strains were archived at -80°C for downstream applications.

**Figure S17. Repair of *ade2-1* and Construction of CRM-driven *ADE2* Strain in W303α.** The same genome engineering strategy and validation procedures described in Figure S16 (Supporting Information) were applied to the W303**α** background. Successfully validated strains were archived at -80°C for downstream applications.

**Figure S18. Synthetic Logic Gate Control of Endogenous Yeast Genes via dCm-mediated DNA Methylation.** The logic gate configuration and *eMEMS::ADE2* control followed the description in Figure 5, except that W303a (top) and W303α (bottom) strains were cultured for 3 days. Data represent undiluted colonies.

**Table S1. Oligos Used in this Study.**

| **Oligo Name** | **Sequence (5’-3’)** | **Purpose/Methodology** |
| --- | --- | --- |
| APT F | cagggacttcagtaggtgggtgtag | Removing the *Aar*I site in APT through synonymous mutation using Snap assembly (Figure S1, Supporting Information) |
| APT R | ctacacccacctactgaagtccctg |  |
| SacB-GmR F | gacaacatgtcgaggctcagcagaacctcctgcaggtgcctcacatatacctgccgttc | Amplifying the *sacB-GmR* cassette from pLZ-sacB and inserting it into pEarlyGate303 (pGW303) through the *Sbf* I site using Snap assembly (Figure S1 and Table S2, Supporting Information) |
| SacB-GmR R | gtaaaacgacggccagtgccaactaggcatgcaggtgggcgcaatcacgaatgaataac |  |
| AtUBQ1 Pro F | ttcacctgcagagAACCggtacccccgggatatttcacaaattg | Constructing CRISPR-coupled *Epi*-effector expression vector (Figure S1, Supporting Information). Specifically, amplifying the *AtUBQ1* promoter from pMDC99, the N and C termini of Cas9 from pFGC-pcoCas9 (Addgene), TET1cd from pUBQ10::ZF108 3xFLAG TET1cd (Addgene), and the *AtUBQ1* terminator from pMDC99, respectively. *DRM2* and *DRM2cd* as effectors were amplified from the cDNA of *A. thaliana*. These fragments were inserted into the pJET1.2 cloning vector using Blunt ligation. All intermediate pJET1.2 vectors were integrated into pGW303-mPAT (Figure S1 and Table S2, Supporting Information) via Golden Gate technology. A TET1cd-centered dCd sequence is shown in Supplemental Sequence Note 1 |
| AtUBQ1 Pro R | ttcacctgcagagGGGAtttgtgtttcgtctctctcac |  |
| N-Cas9 F | ttcacctgcagagTCCCatggattacaaagacgatgacgataaggattacaaggatgatgatgataaggattacaaggatgatgatgataag |  |
| N-Cas9 R | ttcacctgcagagCAGActgcttatctctgattc |  |
| C-Cas9 F | ttcacctgcagagTCTGgaaagaccatccttgatttcttg |  |
| C-Cas9 R | ttcacctgcagagCTTCttcttcttagcctgtccagc |  |
| TET1cd F | ttcacctgcagagGAAGgctagggacggatccctgccgacctgcagctg |  |
| TET1cd R | ttcacctgcagagTCAGacccaatggttatagggccc |  |
| DRM2 F | cacctgcagagGAAGgctagggacgtgatttggaataacgatgatgatgattttttggag |  |
| DRM2cd F | cacctgcagagGAAGgctagggacagatcatctgttgatgacgagccgattc |  |
| DRM2 R | cacctgcagagTCAGtcaagatcctctcatcctcgcacgtacc |  |
| AtUBQ1 Ter F | ttcacctgcagagCTGAggatccagagactcttatc |  |
| AtUBQ1 Ter R | ttcacctgcagagCTAGcctgcaggacataaacggtcattatttc |  |
| TET1cd-M F | ggacttctgtgctcatccctacagggccattcacaacatgaataatgg | Producing mutated TET1cd and dead Cas9 using site-directed mutagenesis technology in the intermediate pJET1.2 vector |
| TET1cd-MR | ccattattcatgttgtgaatggccctgtagggatgagcacagaagtcc |  |
| H840A F | ctgattacgatgttgatgcaatcgttccacagtc |  |
| H840A R | gactgtggaacgattgcatcaacatcgtaatcag |  |
| D10A F | gtactctatcggacttgcaatcggaaccaactc |  |
| D10A R | gagttggttccgattgcaagtccgatagagtac |  |
| eMEMS F | ttcacctgcagagAACCggctgtacagagaccgttagttcatataattttaaa | Constructing a CRM reporting system used in plants (Figure 1; Figure S1 Supporting Information). Specifically, amplifying the eMEMS and LAP1 elements from *A. thaliana* genomic DNA, the luciferase gene with and without mini *35S* promoter from pCambia1301-Luc system, the MAS terminator from pEarleyGate 103, respectively. These fragments were inserted into the pJET1.2 cloning vector using Blunt ligation. All intermediate pJET1.2 vectors were integrated into pLZENTR-AARI (Figure S1 and Table S2, Supporting Information) via Golden Gate technology, along with direct annealed linker and pLZ-Donors expressing relevant gRNAs (Figure S1 Supporting Information). An example sequence is shown in Supplemental Sequence Note 3, along with explanations of other reporters |
| eMEMS R | ttcacctgcagagGCTGaccagagcacgacagtctatgtagggcgaaagttcgtttgg |  |
| LAP1 F | ttcacctgcagagAACCggctgtacagagacccgagaaccattccgaacccg |  |
| LAP1 R | ttcacctgcagagGCTGaccagagcacgacagtctatgtgttagtgagtagctcaattagg |  |
| Luc + 35S Mini F | ttcacctgcagagCAGCgcaagacccttcctctatataaggaagttcatttcatttggagaggacacccggcgcatcaatggaagacgccaaaaac |  |
| Luc - 35S Mini F | ttcacctgcagagCAGCcccggcgcatcaatggaagacgccaaaaac |  |
| Luc R | ttcacctgcagagCACTtccagcgtaatctggaacatcgtatgggtatctagatccagacaatttggactttccgccc |  |
| Linker F | AGTGgtgagggcagaggaagttga |  |
| Linker R | TGTAtcaacttcctctgccctcac |  |
| MAS Ter F | ttcacctgcagagTACAgaatcttggactcccatgttggc |  |
| MAS Ter R | ttcacctgcagagGAGAgataatttatttgaaaattcataagaaaag |  |
| ADH1 Pro F | aaaccgaaatcaaaaaaaagaataaaaaaaaaatgatgaattgaaaagcttatccttttgttgtttccg | Constructing a DF reporting system used in yeast (Figure 2; Figure S2 and Table S2, Supporting Information). Specifically, amplifying the *ADH1* promoter and terminator from pGADT7-GW and *mRuby2* from pFA6a-link-yomRuby2-SpHis5 (Addgene). These elements were then inserted into the pLZ-AbAi-GW at the *Hind* III and *Kpn* I sites using Snap assembly. Amplifying *Clover* from pFA6a-link-yoClover-SpHis5 (Addgene), then inserting it into the pLZ-AbAi-GW at the *Xho* I and *Afl* II sites using Snap assembly (Figure S2 Supporting Information) |
| ADH1 Pro R | actcctctcctttggacaccatctttcaggaggcttgcttcaagcttggagttgattgtatgcttggta |  |
| mRuby2 F | taccaagcatacaatcaactccaagcttgaagcaagcctcctgaaagatggtgtccaaaggagaggagt |  |
| mRuby2 R | aaaatcataaatcataagaaattcgcttatttagaagtggcgcgccttacttatacaattcatccatac |  |
| ADH1 Ter F | gtatggatgaattgtataagtaaggcgcgccacttctaaataagcgaatttcttatgatttatgatttt |  |
| ADH1 Pro R | ctcgttcagcttttttgtacaaacttgtttgatagcttggcgcgccgccggtagaggtgtggtcaat |  |
| His-Clover F | tcgatgggggatctgtcgacctcgagtctagaaattcctggcattatcacataatgaattatacattatataaagtaatgtgatttcttcgaagaatatactaaaaaatgagcaggcaagataaacgaaggcaaagatggtttctaaaggtgaag |  |
| His-Clover R | gtgttttttaaatagtacataatggatttccttaagttacgcggctgtgacaaattc |  |
| Y-eMEMS F | ttcacctgcagagAACCgttagttcatataattttaaa | eMEMS, LAP1, and ROM1 were amplified from the Col-0 ecotype, cloned into the intermediate vector pLZENTR-AARI, and subsequently recombined into pLZ-AbAi-GW (Table S2). The equivalent eMEMS sequences from the C24 ecotype or *Arabidopsis. lyrata* were directly amplified from their respective genomic DNA and subjected to the same cloning strategy. All resulting reporter constructs were used in yeast. |
| Y-eMEMS R | ttcacctgcagagCTAGgtagggcgaaagttcgtttgg |  |
| Y-LAP1 F | ttcacctgcagagAACCcgagaaccattccgaacccg |  |
| Y-LAP1 R | ttcacctgcagagCTAGgttagtgagtagctcaattag |  |
| Y-ROM1 F | ttcacctgcagagAACCtttccatccacttcccaaaaggttc |  |
| Y-ROM1 R | ttcacctgcagagCTAGtttgactaagggcttgactcg |  |
| C24 eMEMS F | ttcacctgcagagAACCgttagttcatataatttcgaaatag |  |
| C24 eMEMS R | ttcacctgcagagCTAGgtagcgcgaaagttcgtttgg |  |
| A. lyrata eMEMS F | ttcacctgcagagAACCaattagttagtgcatataatttc |  |
| A. lyrata eMEMS R | ttcacctgcagagCTAGgtagcgccaaagttcgtttgg |  |
| dAcrIIA1 F | ggggacaagtttgtacaaaaaagcaggcttcatgaccatcaagttgctcgacg | Amplifying *AcrIIA1* from pJH372 (Addgene) and integrating it into pDONR207 |
| dAcrIIA1 R | ggggaccactttgtacaagaaagctgggtcaagcagctcattttttttttccttcag |  |
| dAcrIIA2 F | ggggacaagtttgtacaaaaaagcaggcttcatgacgctgacccgcgctcag | Amplifying *AcrIIA2* from pJH373 (Addgene) and integrating it into pDONR207 |
| dAcrIIA2 R | ggggaccactttgtacaagaaagctgggtccttcagttcggacttaagaataatc |  |
| dAcrIIA3 F | ggggacaagtttgtacaaaaaagcaggcttcatgacaaagtataataagtcag | Amplifying *AcrIIA3* from pJH375 (Addgene) and integrating it into pDONR207 |
| dAcrIIA3 R | ggggaccactttgtacaagaaagctgggtcagcaagaccgagcttcacatacag |  |
| dAcrIIA4 F | ggggacaagtttgtacaaaaaagcaggcttcatgaacattaacgacctcatac | Amplifying *AcrIIA4* from pJH376 (Addgene) and integrating it into pDONR207 |
| dAcrIIA4 R | ggggaccactttgtacaagaaagctgggtcgttcagttcacttttcaacgt |  |
| dAcrIIA4-LOV2 F | ggggacaagtttgtacaaaaaagcaggcttcatgaacattaacgacctgattc | Amplifying *AcrIIA4-LOV2* from CMV-CASANOVA (Addgene) and integrating it into pDONR207 |
| dAcrIIA4-LOV2 R | ggggaccactttgtacaagaaagctgggtcgttcagctcagatttcagtgtg |  |
| dAcrIIC1Nme F | ggggacaagtttgtacaaaaaagcaggcttcatgaataaaacttataaaattgg | Amplifying *AcrIIC1Nme* from pEJS433-pCSDest2-AcrIIC1Nme (Addgene) and integrating it into pDONR207 |
| dAcrIIC1Nme R | ggggaccactttgtacaagaaagctgggtctagttcaacaaactcccaacatg |  |
| dAcrIIC1Boe F | ggggacaagtttgtacaaaaaagcaggcttcatgaaagaggtatttaaattaaaac | Amplifying A*crIIC1Boe* from pEJS430-pCSDest2-AcrIIC1Boe (Addgene) and integrating it into pDONR207 |
| dAcrIIC1Boe R | ggggaccactttgtacaagaaagctgggtccattacattgaattcccaacac |  |
| dAcrIIC2 F | ggggacaagtttgtacaaaaaagcaggcttcatgagcaaaaacaatattttcaac | Amplifying A*crIIC2* from pEJS436-pCSDest2-AcrIIC2Nme (Addgene) and integrating it into pDONR207 |
| dAcrIIC2 R | ggggaccactttgtacaagaaagctgggtcatcatcccagccctccaaatccg |  |
| dAcrIIC3 F | ggggacaagtttgtacaaaaaagcaggcttcatgttcaaacgcgctattatcttc | Amplifying AcrIIC3 from pEJS482-pCDest2-AcrIIC3Nme-mTagBFP2-IRES (Addgene) and integrating it into pDONR207 |
| dAcrIIC3 R | ggggaccactttgtacaagaaagctgggtcaattttaaaaatctctttgccgttggc |  |
| 35S gRNA1 F | cacgaagcctctctaaccatctgt | *35S* promoter gRNAs were annealed and inserted into the pLZ-Donor I, pLZ-Donor II, and pLZ-Donor III, respectively, via the *Bsa* I sites. These constructs were subsequently integrated into the dCd system collectively |
| 35S gRNA1 R | aaacacagatggttagagaggctt |  |
| 35S gRNA2 F | attgtcagaagtactattccagta |  |
| 35S gRNA2 R | aaactactggaatagtacttctga |  |
| 35S gRNA3 F | attgacagaactcgccgtaaagac |  |
| 35S gRNA3 R | aaacgtctttacggcgagttctgt |  |
| eMEMS gRNA1 F | attgactaacggtctctgtacagc | eMEMS target gRNAs were annealed and inserted separately into pLZ-Donor II vector via the *Bsa* I sites. These constructs were subsequently integrated into the dCd system individually. |
| eMEMS gRNA1 R | aaacgctgtacagagaccgttagt |  |
| eMEMS gRNA2 F | attgttctacaaaatctcctagactat |  |
| eMEMS gRNA2 R | aaacatagtctaggagattttgtagaa |  |
| eMEMS gRNA3 F | attgctacatagactgtcgtgctc |  |
| eMEMS gRNA3 R | aaacgagcacgacagtctatgtag |  |
| Y-eMEMS gRNA F | gatcttctacaaaatctcctagactat | eMEMS target gRNA was annealed and inserted into pLZ-Y-Donor II vector via the *Bsa* I sites |
| Y-eMEMS gRNA R | aaacatagtctaggagattttgtagaa |  |
| IBM1 gRNA1 F | cacgaagatcggtcattgccgaat | *IBM1* target gRNAs were annealed and inserted separately into pLZ-Donor II via the *Bsa* I sites. These constructs were subsequently integrated into the dCd system individually. |
| IBM1 gRNA1 R | aaacattcggcaatgaccgatctt |  |
| IBM1 gRNA2 F | cacgagttccagttctggcaactag |  |
| IBM1 gRNA2 R | aaacctagttgccagaactggaact |  |
| IBM1 gRNA3 F | cacgagggatgttcaatcaggattt |  |
| IBM1 gRNA3 R | aaacaaatcctgattgaacatccct |  |
| TK gRNA1 F | gatcgggggtgcttaagaggatcg | TK target gRNAs were annealed and inserted into pLZ-Y-Donor (I, II, and III) vectors via the *Bsa* I sites. These constructs were subsequently integrated into the dCm system separately. |
| TK gRNA1 R | aaaccgatcctcttaagcaccccc |  |
| TK gRNA2 F | gatcgtacactatcttgtcacccgg |  |
| TK gRNA2 R | aaacccgggtgacaagatagtgtac |  |
| TK gRNA3 F | gatcgttaatatgcgaagtggacct |  |
| TK gRNA3 R | aaacaggtccacttcgcatattaac |  |
| LAP1 gRNA F | gatctggatttgtagggtaagaca | Target gRNAs of positive (LAP1) and negative (ROM1) controls were annealed and inserted into pLZ-Y-Donor II vector via the Bsa I sites. Along with the gRNA for an unknown protein (another negative control), these gRNA sequences were used as sc gRNA for eMEMS and TK both in planta and in yeast |
| LAP1 gRNA R | aaactgtcttaccctacaaatcca |  |
| ROM1 gRNA F | gatcggtacgaaccttttgggaag |  |
| ROM1gRNA R | aaaccttcccaaaaggttcgtacc |  |
| UP gRNA F | gatccctctctggaaaatgataggtg |  |
| UP gRNA R | aaaccacctatcattttccagagagg |  |
| ROX3 F | ctaaagggaacaaaagctggagctcgcgtattaccttctgctgga | Amplifying the *ROX3* promoter from BY4742 and replacing the GAP promoter in pAG424GAP-ccdB-HA via the *Sac* I and *Spe* I sites using Snap assembly (Figure 2 and Table S2, Supporting Information) |
| ROX3 R | caaacttgtgatgggggatccactagatgtattatcgtattcctttttc |  |
| dTk F | ggggacaagtttgtacaaaaaagcaggcttcaatgagtcatcggacctcgcgg | Amplifying the TK promoter from pRL-HSV TK-Renilla Luc (Promega) and cloning into pDONR207 using Gateway technology |
| dTk R | ggggaccactttgtacaagaaagctgggtcttaagcgggtcgctgcaggg |  |
| Actin 8 qF | cttaccgaggctcctcttaaccc | Detecting *SUC2* expression by Real-time PCR |
| Actin 8 qR | cagagtccaacacaataccggttg |  |
| UBQ5 qF | gacgcttcatctcgtcc |  |
| UBQ5 qR | gtaaacgtaggtgagtcca |  |
| SUC2 qF | tagccattgtcgtccctca |  |
| SUC2 qR | ccaccaccgaatagttcgtc |  |
| Set1-Kan F | cattccttatttgttgaatctttataagaggtctctgcgtttagagacggatccccgggttaattaag | Amplifying the KanMx expression cassette from pLZ-14252-KanMX (Table S2) and transforming PCR products into yeast in the W303a/**α** backgrounds for replacement of the coding regions of *Set1*, *Set2*, and *SWD1* using homologous recombination. |
| Set1-Kan R | caacgatatgttaaatcaggaagctccaaacaaatcaatgtatcatcgcgaattcgagctcgttttcg |  |
| Set2-Kan F | tcgtgctgtcaaacctttctcctttcctggttgttgttttacgtgatccggatccccgggttaattaag |  |
| Set2-Kan R | tttgggacagaaaacgtgaaacaagccccaaatatgcatgtctggttaacgaattcgagctcgttttcg |  |
| SWD1-Kan F | cgagaccgagtccttttagtttgtgtatatcagctggttcttttcgttcggatccccgggttaattaag |  |
| SWD1-Kan R | ctcttcagtgtcagttgcctatttaaactttctccttttgaagagaaaccgaattcgagctcgttttcg |  |
| 35S 1st Bis-F1 | gttgtaaaaygayggttagtgttaagtttgtatgt | Amplifying the bisulfate-converted genomic DNA for Wide-seq using nest-PCR |
| 35S 1st Bis-R1 | caaatatatcrtactccaccatattaacraaaattttc |  |
| 35S 2nd Bis-F1 | gtttatatagagttttttaygatttaatgat |  |
| 35S 2nd Bis-R1 | tatcttcrtaacacraaatcctctaaaa |  |
| 35S 1st Bis-F2 | ggttagtgttaagtttgtatgtttgtaggttt |  |
| 35S 1st Bis-R2 | tactccaccatattaacraaaattttcttcttatcatta |  |
| 35S 2nd Bis-F2 | ttargatttaatgataagaagaaaattttygttaat |  |
| 35S 2nd Bis-R2 | cacraaatcctctaaaatcccccrtattc |  |
| 3 x LAP1 F1 | aggattattCACCTGCagagAACCcgagaaccattccgaacccg | Amplifying the fragments of LAP1 or eMEMS from the Col-0 genome and cloning them into pLZENTR-AARI (Table S2) in three copies via Golden Gate assembly. The resulting constructs were then integrated into the DF reporter system (Figure S2 and Table S2, Supporting Information). All final constructs were used in yeast. |
| 3 x LAP1 R1 | aggattattCACCTGCagagccgtgtcgttagtgagtagctcaattagg |  |
| 3 x LAP1 F2 | aggattattCACCTGCagagacggcgagaaccattccgaacccg |  |
| 3 x LAP1 R2 | aggattattCACCTGCagagtatggttagtgagtagctcaattaggc |  |
| 3 x LAP1 F3 | aggattattCACCTGCagagcatatggcgagaaccattccgaacccg |  |
| 3 x LAP1 R3 | aggattattCACCTGCagagCTAGgttagtgagtagctcaattaggcttg |  |
| 3 x eMEMS F1 | aggattattCACCTGCagagAACCgttagttcatataattttaaatag |  |
| 3 x eMEMS R1 | aggattattCACCTGCagagccgtgtcgtagggcgaaagttcgtttgg |  |
| 3 x eMEMS F2 | aggattattCACCTGCagagacgggttagttcatataattttaaatag |  |
| 3 x eMEMS R2 | aggattattCACCTGCagag  tatggtagggcgaaagttcgtttggttg |  |
| 3 x eMEMS F3 | aggattattCACCTGCagagcatatgggttagttcatataattttaaat |  |
| 3 x eMEMS R3 | aggattattCACCTGCagagCTAGgtagggcgaaagttcgtttggttg |  |
| Solyc01g008790F1 | cacctgcagagaacctgctcgtattgaatttcccacg | Amplifying the fragment of *Solyc01g008790* from the tomato genome and cloning it into pLZENTR-AARI (Table S2) in three copies via Golden Gate assembly, which was then integrated into pLZ-AbAi-GW. |
| Solyc01g008790R1 | cacctgcagagccgttgtatgtccatggatatggtgttgtttgtctatc |  |
| Solyc01g008790F2 | cacctgcagagacggctggagtgtactgctcgtattgaatttcccac |  |
| Solyc01g008790R2 | cacctgcagagtatgtcagactcctcgatatggtgttgtttgtctatcg |  |
| Solyc01g008790F3 | cacctgcagagcataggaggtacgtgtgctcgtattgaatttcccacg |  |
| Solyc01g008790R3 | cacctgcagagctaggatatggtgttgtttgtctatcgg |  |
| Solyc01g095700F1 | cacctgcagagaaccatcatcgtattaaaaaatttatattagagtttg | Amplifying the fragment of *Solyc01g095700* from the tomato genome and cloning it into pLZENTR-AARI (Table S2) in three copies via Golden Gate assembly, which was then integrated into pLZ-AbAi-GW. |
| Solyc01g095700R1 | cacctgcagagccgttgtatgtccatgggaatataataatgaaagcttacgc |  |
| Solyc01g095700F2 | cacctgcagagacggctggagtgtacatcatcgtattaaaaaatttatattagag |  |
| Solyc01g095700R2 | cacctgcagagtatgtcagactcctcggaatataataatgaaagcttacgc |  |
| Solyc01g095700F3 | cacctgcagagcataggaggtacgtgatcatcgtattaaaaaatttatattagag |  |
| Solyc01g095700R3 | cacctgcagagctagggaatataataatgaaagcttacgc |  |
| Solyc07g066080F1 | cacctgcagagaaccatgcgttactgcaggggcggagttagg | Amplifying the fragment of *Solyc07g066080* from the tomato genome and cloning it into pLZENTR-AARI (Table S2) in three copies via Golden Gate assembly, which was then integrated into pLZ-AbAi-GW. |
| Solyc07g066080R1 | cacctgcagagccgttgtatgtccatgacaggaaaaaacaaatacacaaaagaag |  |
| Solyc07g066080F2 | cacctgcagagacggctggagtgtacatgcgttactgcaggggcggagttagg |  |
| Solyc07g066080R2 | cacctgcagagtatgtcagactcctcacaggaaaaaacaaatacacaaaagaag |  |
| Solyc07g066080F3 | cacctgcagagcataggaggtacgtgatgcgttactgcaggggcggagttagg |  |
| Solyc07g066080R3 | cacctgcagagctagacaggaaaaaacaaatacacaaaagaagtgacg |  |
| Solyc09g091470F1 | cacctgcagagaaccacgatgacgtgtggaattcaactagcg | Amplifying the fragment of *Solyc09g091470* from the tomato genome and cloning it into pLZENTR-AARI (Table S2) in three copies via Golden Gate assembly, which was then integrated into pLZ-AbAi-GW. |
| Solyc09g091470R1 | cacctgcagagccgttgtatgtccatggggagctacatgacaaatctttcg |  |
| Solyc09g091470F2 | cacctgcagagacggctggagtgtacacgatgacgtgtggaattcaactag |  |
| Solyc09g091470R2 | cacctgcagagtatgtcagactcctcgggagctacatgacaaatctttcg |  |
| Solyc09g091470F3 | cacctgcagagcataggaggtacgtgacgatgacgtgtggaattcaactag |  |
| Solyc09g091470R3 | cacctgcagagctaggggagctacatgacaaatctttcg |  |
| Tomato gRNA1 F | gatcagcgatgttctgactttgaa | *Solyc01g008790* target gRNA inserted into pLZ-Y-Donor II via the *Bsa* I sites |
| Tomato gRNA1 R | aaacttcaaagtcagaacatcgct |  |
| Tomato gRNA2 F | gatcaaagcttacgcaaaaatcagt | *Solyc01g095700* target gRNA inserted into pLZ-Y-Donor II via the *Bsa* I sites |
| Tomato gRNA2 R | aaacactgatttttgcgtaagcttt |  |
| Tomato gRNA3 F | gatcaacccaccgtttcgtttatgt | *Solyc07g066080* target gRNA inserted into pLZ-Y-Donor II via the *Bsa* I sites |
| Tomato gRNA3 R | aaacacataaacgaaacggtgggtt |  |
| Tomato gRNA4 F | gatcatatcaggaaaaaattaatat | *Solyc09g091470* target gRNA inserted into pLZ-Y-Donor II via the *Bsa* I sites |
| Tomato gRNA4 R | aaacatattaattttttcctgatat |  |
| SSO1 gRNA1 F | gatctatgcaggctgtgcgcatgg | *SSO1* and *SSO2* target gRNAs inserted into pLZ-Y-Donor II via the *Bsa* I sites |
| SSO1 gRNA1 R | aaacccatgcgcacagcctgcata |  |
| SSO1 gRNA2 F | gatccaaatcgtggaagttacaag |  |
| SSO1 gRNA2 R | aaaccttgtaacttccacgatttg |  |
| SSO2 gRNA1 F | gatccgggtaccaagcggcagcaa |  |
| SSO2 gRNA1 R | aaacttgctgccgcttggtacccg |  |
| SSO2 gRNA2 F | gatcgataagcacgtcgtttaggg |  |
| SSO2 gRNA2 R | aaacccctaaacgacgtgcttatc |  |
| C24 gRNA F | gatcgctacaaagtcttctagacta | gRNAs targeting eMEMS derived from C24 or *A. lyrata*, inserted into pLZ-Y-Donor II via *Bsa* I sites |
| C24 gRNA R | aaactagtctagaagactttgtagc |  |
| A. lyrata gRNA F | gatcgtacaaagtctcctggactat |  |
| A. lyrata gRNA R | aaacatagtccaggagactttgtac |  |
| ADE2 correct F | gatatcgaaaaactagctgaaaaatg | Amplifying the wild-type fragments of ADE2 or URA3 from corresponding plasmids and transforming the PCR products into yeast to repair the endogenous *ade2-1* or *ura3-1* mutant loci |
| ADE2 correct R | agtccggaactctagcaggc |  |
| URA3 correct F | catgtcgaaagctacatataagg |  |
| URA3 correct R | gctggccgcatcttctcaaatatg |  |
| URA3 gRNA1 F | gatcttcggtaatctccgagcaga | gRNAs targeting *ADE2* or *URA3* locus in the repaired yeast, inserted into pLZ-Y-Donor II via *Bsa* I sites |
| URA3 gRNA1 R | aaactctgctcggagattaccgaa |  |
| URA3 gRNA2 F | gatcccgagcagaaggaagaacga |  |
| URA3 gRNA2 R | aaactcgttcttccttctgctcgg |  |
| URA3 gRNA3 F | gatcgaaggagcacagacttagat |  |
| URA3 gRNA3 R | aaacatctaagtctgtgctccttc |  |
| ADE2 gRNA1 F | gatcgacaaatgactcttgttgta |  |
| ADE2 gRNA1 R | aaactacaacaagagtcatttgtc |  |
| ADE2 gRNA2 F | gatccttgttgtagggctacgaac |  |
| ADE2 gRNA2 R | aaacggtaatactaagtgattgac |  |
| ADE2 gRNA3 F | gatcgttcgtagccctacaacaag |  |
| ADE2 gRNA3 R | aaactaatattgtccatttagttc |  |
| ADE2 KanMX F | caagaaaatcggacaaaacaatcaagtacgacatggaggcccagaataccc | Amplifying the KanMX expression cassette from pLZ-14252-KanMX, and ROM1, eMEMS, and LAP1 from the corresponding pLZ-AbAi-GW plasmids. Overlap PCR was used to fuse the fragments, and the resulting products were transformed into the repaired yeast strains to generate ROM1-, eMEMS-, or LAP1-driven *URA3⁺* or *ADE2⁺* cells |
| ROM1 KanMX R | gtacgaaccttttgggaagtggatggaaacagtatagcgaccagcattcacatac |  |
| KanMX ROM1 F | gtatgtgaatgctggtcgctatactgtttccatccacttcccaaaaggttcgtac |  |
| ADE2 R | ctaatataccaactgttctagaatccattattaatttagtgtgtgtatttgtg |  |
| eMEMS KanMX R | caaccaaacgaactttcgccctaccagtatagcgaccagcattcacatac |  |
| KanMX eMEMS F | gtatgtgaatgctggtcgctatactggtagggcgaaagttcgtttggttg |  |
| LAP1 KanMX R | gatttatacgggttcggaatggttctcgcagtatagcgaccagcattcacatac |  |
| KanMX LAP1 F | gtatgtgaatgctggtcgctatactgcgagaaccattccgaacccgtataaatc |  |
| URA3 KanMX F | GAACAAAAACCTGCAGGAAACGAAGATAAATCacGACATGGAGGCCCAGAATACCC |  |
| URA3 R | cacgttccttatatgtagctttcgacatTATTAATTTAGTGTGTGTATTTGTG |  |
| KanMX seq F | gcagtttcatttgatgctcgatg | Primers used for PCR or sequencing validation during the generation of repaired yeast or CRM-driven *URA3⁺* or *ADE2⁺* cells |
| ADE2 seq F | gctcaacattaagacggtaatac |  |
| ADE2 seq R | gaggccttggggcaatttcg |  |
| URA3 pro seq F | gaacgaaggaaggagcacagac |  |
| URA3 seq R | gaaatcattacgaccgagattccc |  |
| MQ1 inert adaptor | aaccgcaagatgctagagtctaccctca | Adaptors used in AND gate construction |
| gRNA inert adaptor | gtacgcaagatgctagagtctaccctag |  |

**Table S2. Plasmids Used and Created in this Study.**

| **Plasmid Name** | **Details** | **Source** |
| --- | --- | --- |
| pGW303 | pEarleyGate 303/^[7]^ | Pikaard lab |
| pGW303 No *Aar*I | Removing  *Aar*I site in PAT of pGW303/Figure S1, Supporting Information | This study |
| pGW303-mPAT | pGW303 No AarI inserted with the *sacB-GmR* cassette flaked with *Aar*I sites/Figure S1, Supporting Information | This study |
| dCd-UBQ1-dpoCas9-TET1cd | pGW303-mPAT with inserted *AtUBQ1* pro, dpoCas9, and TET1cd comprising a dCd system/Supplemental Sequence Note 1 | This study |
| dCd-UBQ1-dpoCas9-TET1cd-M | pGW303-mPAT with the inserted *AtUBQ1* pro, dpoCas9, and mutated TET1cd comprising a dCd control | This study |
| dCd-UBQ1-dpoCas9-TET1cdΔ | pGW303-mPAT with the inserted *AtUBQ1* pro, dpoCas9, and deleted TET1cd comprising the other dCd control | This study |
| dCm-UBQ1-dpoCas9-DRM2 | pGW303-mPAT with the inserted *AtUBQ1* pro, dpoCas9, and DRM2 comprising a dCm system | This study |
| dCm-UBQ1-dpoCas9-DRM2cd | pGW303-mPAT with the inserted *AtUBQ1* pro, dCas9, and DRM2cd comprising a dCm system | This study |
| pLZ-sacB | A self-designed cloning vector with a *sacB* cassette flanked by *Bbs* I and *Sap* I sites, along with the *Gentamycin Acetyltransferase* gene (GmR) | Homemade |
| pLZ-Donor I | *AtU3b*-driven cassette inserted into pLZ-sacB as donor I for gRNA/Supplemental Sequence Note 2 | This study |
| pLZ-Donor II | *AtU6_1*-driven cassette inserted into pLZ-sacB as donor II for gRNA/Supplemental Sequence Note 2 | This study |
| pLZ-Donor III | *AtU6_26*-driven cassette inserted into pLZ-sacB as donor III for gRNA/Supplemental Sequence Note 2 | This study |
| pLZ-Donor IV | *AtU6_29*-driven cassette inserted into pLZ-sacB as donor IV for gRNA/Supplemental Sequence Note 2 | This study |
| pLZ-Adaptor I | A short fragment in pLZ-sacB to fill gap from unused pLZ-Donor I/Supplemental Sequence Note 2 | This study |
| pLZ-Adaptor II | A short fragment in pLZ-sacB to fill gap from unused pLZ-Donor II/Supplemental Sequence Note 2 | This study |
| pLZ-Adaptor III | A short fragment in pLZ-sacB to fill gap from unused pLZ-Donor III/Supplemental Sequence Note 2 | This study |
| pLZ-Adaptor IV | A short fragment in pLZ-sacB to fill gap from unused pLZ-Donor IV/Supplemental Sequence Note 2 | This study |
| pLZ-Y-Donor I | *SNR52*-driven cassette inserted into pLZ-sacB as donor I for gRNA delivery in yeast/Supplemental Sequence Note 5 | This study |
| pLZ-Y-Donor II | *SNR52*-driven cassette inserted into pLZ-sacB as donor II for gRNA delivery in yeast/Supplemental Sequence Note 5 | This study |
| pLZ-Y-Donor III | *SNR52*-driven cassette inserted into pLZ-sacB as donor III for gRNA delivery in yeast/Supplemental Sequence Note 5 | This study |
| pLZ-Y-Adaptor I | A short fragment in pLZ-sacB to fill gap from unused pLZ-Y-Donor I/Supplemental Sequence Note 5 | This study |
| pLZ-Y-Adaptor II | A short fragment in pLZ-sacB to fill gap from unused pLZ-Y-Donor II/Supplemental Sequence Note 5 | This study |
| pLZ-Y-Adaptor III | A short fragment in pLZ-sacB to fill gap from unused pLZ-Y-Donor III/Supplemental Sequence Note 5 | This study |
| pLZENTR-AARI | pENTR™ 1A Dual Selection Vector with custom-modified *Aar*I sites/Figure S1 and S2, Supporting Information | Homemade |
| p14248-GAP | pAG424GAP-ccdB-HA, an original vector with *GAP* promoter | Addgene |
| dCm-GAP-dyopCas9-MQ1 | p14248-GAP with inserted dyopCas9, MQ1, P2A, and Hyg^R^ as a dCm system used in yeast/Supplemental Sequence Note 4 | This study |
| p14248-ROX3 | The GAP promoter of p14248-GAP was replaced with ROX3 promoter to generated pLZ424ROX3-ccdB-HA/Figure S2, Supporting Information | This study |
| dCm-ROX3-dyopCas9-MQ1 | p14248-ROX3 with inserted dyopCas9, MQ1, P2A, and Hyg^R^ as a dCm system used in yeast | This study |
| pmRuby2 | pFA6a-link-yomRuby2-SpHis5 | Addgene |
| pClover | pFA6a-link-yoClover-SpHis5 | Addgene |
| pLZ-AbAi-GW | pAbAi was modified to contain a *ccd*B cassette/Figure S2, Supporting Information | Homemade |
| pLZ-eMEMS-AbA^R^ reporter | eMEMS was integrated into pLZ-AbAi-GW to form the eMEMS-AbA^R^ reporter used in yeast | This study |
| pLZ-C24-eMEMS-AbA^R^ reporter | The equivalent eMEMS derived from the C24 ecotype was integrated into pLZ-AbAi-GW to form the C24 eMEMS-AbA^R^ reporter used in yeast | This study |
| pLZ-*A. lyrata*-eMEMS-AbA^R^ reporter | The equivalent eMEMS derived from the *A. lyrata* was integrated into pLZ-AbAi-GW to form the *A. lyrata* eMEMS-AbA^R^ reporter used in yeast | This study |
| pLZ-TK-AbA^R^ reporter | TK fragment was integrated into pLZ-AbAi-GW to form TK-AbA^R^ reporter used in yeast | This study |
| pLZ-LAP1-AbA^R^ reporter | LAP1 fragment was integrated into pLZ-AbAi-GW to form a positive control used in yeast | This study |
| pLZ-ROM1-AbA^R^ reporter | ROM1 fragment was integrated into pLZ-AbAi-GW to form a negative control used in yeast | This study |
| pLZ-DF-GW | pLZAbAi-GW was modified to contain a DF reporting system: mRuby2 and Clover/Figure S2, Supporting Information | This study |
| pLZ-eMEMS-DF-reporter | eMEMS in three copies was integrated into pLZ-DF-GW to form eMEMS-DF reporter | This study |
| pLZ-LAP1-DF- reporter | LAP1 fragment in three copies was integrated into pLZ-DF-GW to form a positive control | This study |
| p14252 | pAG426GPD-ccdB-HA contains *URA3* selection marker | Addgene |
| pLZ-14252-KanMX | The *URA3* of p14252 is disrupted with an expression cassette of KanMX | Homemade |

**Supplemental Sequence Note 1**

**Detailed Sequence of pGW303-dCd-UBQ1-dpoCas9-TET1cd from Promoter to Terminator Region.** The sequence is color-coded as follows: *AtUBQ1* promoter in green, 3 x FLAG marked in a box, N-terminal NLS in blue, plant codon-optimized Cas9 gene containing the potato IV2 intron (notated with lowercase letters underlined) in grey, C-terminal NLS in blue, TET1cd in orange, *AtUBQ1* terminator in magenta, and landmarks, residues retained after recognition and cleavage by *Aar*I for Golden Gate assembly, shaded in yellow at specific positions throughout the sequence.

AACCGGTACCCCCGGGATATTTCACAAATTGAACATAGACTACAGAATTTTAGAAAACAAACTTTCTCTCTCTTATCTCACCTTTATCTTTTAGAGAGAAAAAGTTCGACTTCCGGTTGACCGGAATGTATCTTTGTTTTTTTTGTTTTGTAACATATTTCGTTTTCCGATTTAGATCGGATCTCCTTTTCCGTTTTGTCGGACCTTCTTCCGGTTTATCCGGATCTAATAATATCCATCTTAGACTTAGCTAAGTTTGGATCTGTTTTTTGGTTAGCTCTTGTCAATCGCCTCATCATCAGCAAGAAGGTGAAATTTTTGACAAATAAATCTTAGAATCATGTAGTGTCTTTGGACCTTGGGAATGATAGAAACGATTTGTTATAGCTACTCTATGTATCAGACCCTGACCAAGATCCAACAATCTCATAGGTTTTGTGCATATGAAACCTTCGACTAACGAGAAGTGGTCTTTTAATGAGAGAGATATCTAAAATGTTATCTTAAAAGCCCACTCAAATCTCAAGGCATAAGGTAGAAATGCAAATTTGGAAAGTGGGCTGGGCCTTTTGTGGTAAAGGCCTGTAACCTAGCCCAATATTAGCAAAACCCTAGACGCGTACATTGACATATATAAACCCGCCTCCTCCTTGTTTAGGGTTTCTACGTGAGAGAGACGAAACACAAATCCCATGGATTACAAAGACGATGACGATAAGGATTACAAGGATGATGATGATAAGGATTACAAGGATGATGATGATAAGATGGCTCCAAAGAAGAAGAGAAAGGTTGGAATCCACGGAGTTCCAGCTGCTGATAAGAAGTACTCTATCGGACTTGCAATCGGAACCAACTCTGTTGGATGGGCTGTTATCACCGATGAGTACAAGGTTCCATCTAAGAAGTTCAAGGTTCTTGGAAACACCGATAGACACTCTATCAAGAAGAACCTTATCGGTGCTCTTCTTTTCGATTCTGGAGAGACCGCTGAGGCTACCAGATTGAAGAGAACCGCTAGAAGAAGATACACCAGAAGAAAGAACAGAATCTGCTACCTTCAGGAAATCTTCTCTAACGAGATGGCTAAGGTTGATGATTCTTTCTTCCACAGACTTGAGGAGTCTTTCCTTGTTGAGGAGGATAAGAAGCACGAGAGACACCCAATCTTCGGAAACATCGTTGATGAGGTTGCTTACCACGAGAAGTACCCAACCATCTACCACCTTAGAAAGAAGTTGGTTGATTCTACCGATAAGGCTGATCTTAGACTTATCTACCTTGCTCTTGCTCACATGATCAAGTTCAGAGGACACTTCCTTATCGAGGGAGACCTTAACCCAGATAACTCTGATGTTGATAAGTTGTTCATCCAGCTTGTTCAGACCTACAACCAGCTTTTCGAGGAGAACCCAATCAACGCTTCTGGAGTTGATGCTAAGGCTATCCTTTCTGCTAGACTTTCTAAGTCTCGTAGACTTGAGAACCTTATCGCTCAGCTTCCAGGAGAGAAGAAGAACGGACTTTTCGGAAACCTTATCGCTCTTTCTCTTGGACTTACCCCAAACTTCAAGTCTAACTTCGATCTTGCTGAGGATGCTAAGTTGCAGCTTTCTAAGGATACCTACGATGATGATCTTGATAACCTTCTTGCTCAGATCGGAGATCAGTACGCTGATCTTTTCCTTGCTGCTAAGAACCTTTCTGATGCTATCCTTCTTTCTGACATCCTTAGAGTTAACACCGAGATCACCAAGGCTCCACTTTCTGCTTCTATGATCAAGAGATACGATGAGCACCACCAGGATCTTACCCTTTTGAAGGCTCTTGTTAGACAGCAGCTTCCAGAGAAGTACAAGGAAATCTTCTTCGATCAGTCTAAGAACGGATACGCTGGATACATCGATGGAGGAGCTTCTCAGGAGGAGTTCTACAAGTTCATCAAGCCAATCCTTGAGAAGATGGATGGAACCGAGGAGCTTCTTGTTAAGTTGAACAGAGAGGATCTTCTTAGAAAGCAGAGAACCTTCGATAACGGATCTATCCCACACCAGATCCACCTTGGAGAGCTTCACGCTATCCTTCGTAGACAGGAGGATTTCTACCCATTCTTGAAGGATAACAGAGAGAAGATCGAGAAGATCCTTACCTTCAGAATCCCATACTACGTTGGACCACTTGCTAGAGGAAACTCTCGTTTCGCTTGGATGACCAGAAAGTCTGAGGAGACCATCACCCCTTGGAACTTCGAGGAGgtaagtttctgcttctacctttgatatatatataataattatcattaattagtagtaatataatatttcaaatatttttttcaaaataaaagaatgtagtatatagcaattgcttttctgtagtttataagtgtgtatattttaatttataacttttctaatatatgaccaaaatttgttgatgtgcagGTTGTTGATAAGGGAGCTTCTGCTCAGTCTTTCATCGAGAGAATGACCAACTTCGATAAGAACCTTCCAAACGAGAAGGTTCTTCCAAAGCACTCTCTTCTTTACGAGTACTTCACCGTTTACAACGAGCTTACCAAGGTTAAGTACGTTACCGAGGGAATGAGAAAGCCAGCTTTCCTTTCTGGAGAGCAGAAGAAGGCTATCGTTGATCTTCTTTTCAAGACCAACAGAAAGGTTACCGTTAAGCAGTTGAAGGAGGATTACTTCAAGAAGATCGAGTGCTTCGATTCTGTTGAAATCTCTGGAGTTGAGGATAGATTCAACGCTTCTCTTGGAACCTACCACGATCTTTTGAAGATCATCAAGGATAAGGATTTCCTTGATAACGAGGAGAACGAGGACATCCTTGAGGACATCGTTCTTACCCTTACCCTTTTCGAGGATAGAGAGATGATCGAGGAGAGACTCAAGACCTACGCTCACCTTTTCGATGATAAGGTTATGAAGCAGTTGAAGAGAAGAAGATACACCGGATGGGGTAGACTTTCTCGTAAGTTGATCAACGGAATCAGAGATAAGCAGTCTGGAAAGACCATCCTTGATTTCTTGAAGTCTGATGGATTCGCTAACAGAAACTTCATGCAGCTTATCCACGATGATTCTCTTACCTTCAAGGAGGACATCCAGAAGGCTCAGGTTTCTGGACAGGGAGATTCTCTTCACGAGCACATCGCTAACCTTGCTGGATCTCCAGCTATCAAGAAGGGAATCCTTCAGACCGTTAAGGTTGTTGATGAGCTTGTTAAGGTTATGGGTAGACACAAGCCAGAGAACATCGTTATCGAGATGGCTAGAGAGAACCAGACCACCCAGAAGGGACAGAAGAACTCTCGTGAGAGAATGAAGAGAATCGAGGAGGGAATCAAGGAGCTTGGATCTCAAATCTTGAAGGAGCACCCAGTTGAGAACACCCAGCTTCAGAACGAGAAGTTGTACCTTTACTACCTTCAGAACGGAAGAGATATGTACGTTGATCAGGAGCTTGACATCAACAGACTTTCTGATTACGATGTTGATGCAATCGTTCCACAGTCTTTCTTGAAGGATGATTCTATCGATAACAAGGTTCTTACCCGTTCTGATAAGAACAGAGGAAAGTCTGATAACGTTCCATCTGAGGAGGTTGTTAAGAAGATGAAGAACTACTGGAGACAGCTTCTTAACGCTAAGTTGATCACCCAGAGAAAGTTCGATAACCTTACCAAGGCTGAGAGAGGAGGACTTTCTGAGCTTGATAAGGCTGGATTCATCAAGAGACAGCTTGTTGAGACCAGACAGATCACCAAGCACGTTGCTCAGATCCTTGATTCTCGTATGAACACCAAGTACGATGAGAACGATAAGTTGATCAGAGAGGTTAAGGTTATCACCTTGAAGTCTAAGTTGGTTTCTGATTTCAGAAAGGATTTCCAGTTCTACAAGGTTAGAGAGATCAACAACTACCACCACGCTCACGATGCTTACCTTAACGCTGTTGTTGGAACCGCTCTTATCAAGAAGTACCCAAAGTTGGAGTCTGAGTTCGTTTACGGAGATTACAAGGTTTACGATGTTAGAAAGATGATCGCTAAGTCTGAGCAGGAGATCGGAAAGGCTACCGCTAAGTACTTCTTCTACTCTAACATCATGAACTTCTTCAAGACCGAGATCACCCTTGCTAACGGAGAGATCAGAAAGAGACCACTTATCGAGACCAACGGAGAGACCGGAGAGATCGTTTGGGATAAGGGAAGAGATTTCGCTACCGTTAGAAAGGTTCTTTCTATGCCACAGGTTAACATCGTTAAGAAAACCGAGGTTCAGACCGGAGGATTCTCTAAGGAGTCTATCCTTCCAAAGAGAAACTCTGATAAGTTGATCGCTAGAAAGAAGGATTGGGACCCAAAGAAGTACGGAGGATTCGATTCTCCAACCGTTGCTTACTCTGTTCTTGTTGTTGCTAAGGTTGAGAAGGGAAAGTCTAAGAAGTTGAAGTCTGTTAAGGAGCTTCTTGGAATCACCATCATGGAGCGTTCTTCTTTCGAGAAGAACCCAATCGATTTCCTTGAGGCTAAGGGATACAAGGAGGTTAAGAAGGATCTTATCATCAAGTTGCCAAAGTACTCTCTTTTCGAGCTTGAGAACGGAAGAAAGAGAATGCTTGCTTCTGCTGGAGAGCTTCAGAAGGGAAACGAGCTTGCTCTTCCATCTAAGTACGTTAACTTCCTTTACCTTGCTTCTCACTACGAGAAGTTGAAGGGATCTCCAGAGGATAACGAGCAGAAGCAGCTTTTCGTTGAGCAGCACAAGCACTACCTTGATGAGATCATCGAGCAAATCTCTGAGTTCTCTAAGAGAGTTATCCTTGCTGATGCTAACCTTGATAAGGTTCTTTCTGCTTACAACAAGCACAGAGATAAGCCAATCAGAGAGCAGGCTGAGAACATCATCCACCTTTTCACCCTTACCAACCTTGGTGCTCCAGCTGCTTTCAAGTACTTCGATACCACCATCGATAGAAAAAGATACACCTCTACCAAGGAGGTTCTTGATGCTACCCTTATCCACCAGTCTATCACCGGACTTTACGAGACCAGAATCGATCTTTCTCAGCTTGGAGGAGATAAGAGACCAGCTGCTACCAAGAAGGCTGGACAGGCTAAGAAGAAGAAGGCTAGGGACGGATCCCTGCCGACCTGCAGCTGTCTTGATCGAGTTATACAAAAAGACAAAGGCCCATATTATACACACCTTGGGGCAGGACCAAGTGTTGCTGCTGTCAGGGAAATCATGGAGAATAGGTATGGTCAAAAAGGAAACGCAATAAGGATAGAAATAGTAGTGTACACCGGTAAAGAAGGGAAAAGCTCTCATGGGTGTCCAATTGCTAAGTGGGTTTTAAGAAGAAGCAGTGATGAAGAAAAAGTTCTTTGTTTGGTCCGGCAGCGTACAGGCCACCACTGTCCAACTGCTGTGATGGTGGTGCTCATCATGGTGTGGGATGGCATCCCTCTTCCAATGGCCGACCGGCTATACACAGAGCTCACAGAGAATCTAAAGTCATACAATGGGCACCCTACCGACAGAAGATGCACCCTCAATGAAAATCGTACCTGTACATGTCAAGGAATTGATCCAGAGACTTGTGGAGCTTCATTCTCTTTTGGCTGTTCATGGAGTATGTACTTTAATGGCTGTAAGTTTGGTAGAAGCCCAAGCCCCAGAAGATTTAGAATTGATCCAAGCTCTCCCTTACATGAAAAAAACCTTGAAGATAACTTACAGAGTTTGGCTACACGATTAGCTCCAATTTATAAGCAGTATGCTCCAGTAGCTTACCAAAATCAGGTGGAATATGAAAATGTTGCCCGAGAATGTCGGCTTGGCAGCAAGGAAGGTCGACCCTTCTCTGGGGTCACTGCTTGCCTGGACTTCTGTGCTCATCCCCACAGGGACATTCACAACATGAATAATGGAAGCACTGTGGTTTGTACCTTAACTCGAGAAGATAACCGCTCTTTGGGTGTTATTCCTCAAGATGAGCAGCTCCATGTGCTACCTCTTTATAAGCTTTCAGACACAGATGAGTTTGGCTCCAAGGAAGGAATGGAAGCCAAGATCAAATCTGGGGCCATCGAGGTCCTGGCACCCCGCCGCAAAAAAAGAACGTGTTTCACTCAGCCTGTTCCCCGTTCTGGAAAGAAGAGGGCTGCGATGATGACAGAGGTTCTTGCACATAAGATAAGGGCAGTGGAAAAGAAACCTATTCCCCGAATCAAGCGGAAGAATAACTCAACAACAACAAACAACAGTAAGCCTTCGTCACTGCCAACCTTAGGGAGTAACACTGAGACCGTGCAACCTGAAGTAAAAAGTGAAACCGAACCCCATTTTATCTTAAAAAGTTCAGACAACACTAAAACTTATTCGCTGATGCCATCCGCTCCTCACCCAGTGAAAGAGGCATCTCCAGGCTTCTCCTGGTCCCCGAAGACTGCTTCAGCCACACCAGCTCCACTGAAGAATGACGCAACAGCCTCATGCGGGTTTTCAGAAAGAAGCAGCACTCCCCACTGTACGATGCCTTCGGGAAGACTCAGTGGTGCCAATGCTGCAGCTGCTGATGGCCCTGGCATTTCACAGCTTGGCGAAGTGGCTCCTCTCCCCACCCTGTCTGCTCCTGTGATGGAGCCCCTCATTAATTCTGAGCCTTCCACTGGTGTGACTGAGCCGCTAACGCCTCATCAGCCAAACCACCAGCCCTCCTTCCTCACCTCTCCTCAAGACCTTGCCTCTTCTCCAATGGAAGAAGATGAGCAGCATTCTGAAGCAGATGAGCCTCCATCAGACGAACCCCTATCTGATGACCCCCTGTCACCTGCTGAGGAGAAATTGCCCCACATTGATGAGTATTGGTCAGACAGTGAGCACATCTTTTTGGATGCAAATATTGGTGGGGTGGCCATCGCACCTGCTCACGGCTCGGTTTTGATTGAGTGTGCCCGGCGAGAGCTGCACGCTACCACTCCTGTTGAGCACCCCAACCGTAATCATCCAACCCGCCTCTCCCTTGTCTTTTACCAGCACAAAAACCTAAATAAGCCCCAACATGGTTTTGAACTAAACAAGATTAAGTTTGAGGCTAAAGAAGCTAAGAATAAGAAAATGAAGGCCTCAGAGCAAAAAGACCAGGCAGCTAATGAAGGTCCAGAACAGTCCTCTGAAGTAAATGAATTGAACCAAATTCCTTCTCATAAAGCATTAACATTAACCCATGACAATGTTGTCACCGTGTCCCCTTATGCTCTCACACACGTTGCGGGGCCCTATAACCATTGGGTCTGAGGATCCAGAGACTCTTATCAAGAATCCCATCTCTTGCTTGCTTTTTTTTGTTGCTTCCCTTTGATAGGGTTTGTTTTTCTTGTTTCAGTGACTTTCTATGTTAAAAGATAATGTCAGTAAAAGGATTTGGTTTTCTATTATTCTGAATCGATTACGGAAGATTCTTGCTTAATTCCAATCTATACAAGTATCGTGAAATAATGACCGTTTATGTCCTGCAGGCTAG

**Supplemental Sequence Note 2**

**Partial Sequences of Four Donor and Four Adaptor Vectors Used in Planta, Numbered from A to H.** They were directly synthesized as gBlocks gene fragments or genes and inserted into pLZ-sacB via *Sap* I sites using Goden Gate technology (Table S2).

1. The partial sequence of pLZ-Donor I is color-coded as follows: attL1 in inward orientation in orange, *AtU3b* promoter in green, two back-to-back Bsa I sites marked in a box, optimized gRNA scaffold in blue, *AtU3b* terminator in grey, and attR5 in outward orientation in red. *Aar*I site *italicized* and underlined with landmark shaded in yellow at specific positions throughout the whole sequence.

GGATTATTGCTCTTCGACCCAGTCTTAAGCTCGGGCCCCAAATAATGATTTTATTTTGACTGATAGTGACCTGTTCGTTGCAACAAATTGATGAGCAATGCTTTTTTATAATGCCAACTTTGTACAAAAAAGCAGGCT*CACCTGCATACCTAG*CTACAGTACTTTGGCTTCTTTTCATTTTTGTTTGCAGCTTTTTTTTTTCTTTCTCATATTCCAAATTTGTAATGAAAATTAAACTAATTTCAATGTCTACAACAACTACGTGAAACAACAACAATTTCTGTATTAACCTTTAAATGTTAGTTTACTTTAAATTTTTCTTATGGCTCAGCCTGTGATGGATAACTGAATCAAACAAATGGCGTCTGGGTTTAAGAAGATCTGTTTTGGCTATGTTGGACGAAACAAGTGAACTTTTAGGATCAACTTCCGTTTATATACGGAGCTTATATCGAGCAATAAGATAAGTGGGCTTTTTATGTAATTTAATGGGCTATCGTCCATATATTCACTAATACCCATGCCCAGTACCCATGTATGCGTTTCATATAAGCTCCTAATTTCTCCCACATCGCTCAAATCTAAACAAATCTTGTTGTATATATAACACTGAGGGAGCACCATTGGTCACGAGAGACCGGTCTCAGTTTAAGAGCTATGCTGGAAACAGCATAGCAAGTTTAAATAAGGCTAGTCCGTTATCAACTTGAAAAAGTGGCACCGAGTCGGTGCTTTTTTCTTTTTTTTGCCATAAACTTAAATTTGTATATCGATCATTGTAGATATTGAAAACCTAGAACAAACCAACATCCATGTGAATGTCTTTCATGACTGATTTAGAGATAATTCTTGAATTTTGGAACTAGAATCTATAATGAGCCTAAATTAAAACATTGTGAATGGTCCCATAGATACTTCACTTGTGTGTAGGATCGGCTCTCTTGATGCGAGAATATTCACTTTTGAGAAGAGGAGGGAAGAACGGATTTAGTGGAAATATTTGTGGGCAACACTTTGCATCTATTGCCGACAACGTCGTGCAACGCATTAATTGGTCCAACATTATCAAACTTTCTTGTAAAAACTGATTTATAGATTTCAGTTACGCAACCTTAAACATTGACAAGTGATGACAAACATTTTTTGAGATCGATGGGA*TAACCTGCGCAGGTG*CAACTTTGTATACAAAAGTTGAACGAGAAACGTAAAATGATATAAATATCAATATATTAAATTAGATTTTGCATAAAAAACAGACTACATAATACTGTAAAACACAACATATCCAGTCACTATGAATCAACTACTTAGATGGTATTAGTGACCTGTACTGGACCGAAGAGCAATAATCCT

1. The partial sequence of pLZ-Adaptor I is color-coded as follows: attL1 in inward orientation in orange, stuffer sequence in blue, and attR5 in outward orientation in red. *Aar*I site *italicized* and underlined with landmark shaded in yellow at specific positions throughout the sequence.

GGATTATTGCTCTTCGACCCAGTCTTAAGCTCGGGCCCCAAATAATGATTTTATTTTGACTGATAGTGACCTGTTCGTTGCAACAAATTGATGAGCAATGCTTTTTTATAATGCCAACTTTGTACAAAAAAGCAGGCT*CACCTGCATACCTAG*GCAAGATGCTAGAGTCTACC*TAACCTGCGCAGGTG*CAACTTTGTATACAAAAGTTGAACGAGAAACGTAAAATGATATAAATATCAATATATTAAATTAGATTTTGCATAAAAAACAGACTACATAATACTGTAAAACACAACATATCCAGTCACTATGAATCAACTACTTAGATGGTATTAGTGACCTGTACTGGACCGAAGAGCAATAATCCT

1. The partial sequence of pLZ-Donor II is color-coded as follows: attL5 in inward orientation in orange, *AtU6-1* promoter in green, two back-to-back Bsa I sites marked in a box, optimized gRNA scaffold in blue, *AtU6-1* terminator in grey, and attR4 in outward orientation in red. *Aar*I site *italicized* and underlined with landmark shaded in yellow at specific positions throughout the sequence.

AGGATTATTGCTCTTCGACCCAGTCTTAAGCTCGGGCCCCAAATAATGATTTTATTTTGACTGATAGTGACCTGTTCGTTGCAACAAATTGATGAGCAATGCTTTTTTATAATGCCAACTTTGTATACAAAAGTTG*CACCTGCATACTAAC*TCTCTATTGGACGGTCATTGTTTTAGTTCCACCACGATTATATTTGAAATTTACGTGAGTGTGAGTGAGACTTGCATAAGAAAATAAAATCTTTAGTTGGGAAAAAATTCAATAATATAAATGGGCTTGAGAAGGAAGCGAGGGATAGGCCTTTTTCTAAAATAGGCCCATTTAAGCTATTAACAATCTTCAAAAGTACCACAGCGCTTAGGTAAAGAAAGCAGCTGAGTTTATATATGGTTAGAGACGAAGTAGTGATTGAGAGACCGGTCTCAGTTTAAGAGCTATGCTGGAAACAGCATAGCAAGTTTAAATAAGGCTAGTCCGTTATCAACTTGAAAAAGTGGCACCGAGTCGGTGCTTTTTTTTGGCAAAAATTTTCAGATTTTTTCTTCATCTGTAGATTTCTGGGTTTTTTTTTCCGTTTCGTGAATCATAAGTGAAGTTTTGGATGCAAATCTGCGCGAAAAAAGTTGGACCTGCAATGAGCTTATTTAGATAGCTAAGACAAAGTGATTGGTCCGTTGTTT*CGCTTCACGCAGGTG*CATGATTACGCCAAGCTATCAACTTTGTATAGAAAAGTTGAACGAGAAACGTAAAATGATATAAATATCAATATATTAAATTAGATTTTGCATAAAAAACAGACTACATAATACTGTAAAACACAACATATCCAGTCACTATGAATCAACTACTTAGATGGTATTAGTGACCTGTACTGCAGGGCGGCCGCATTAGGCACCGACCGAAGAGCAATAATCCT

1. The partial sequence of pLZ-Adaptor II is color-coded as follows: attL5 in inward orientation in orange, stuffer sequence in blue, and attR4 in outward orientation in red. *Aar*I site *italicized* and underlined with landmark shaded in yellow at specific positions throughout the sequence.

AGGATTATTGCTCTTCGACCCAGTCTTAAGCTCGGGCCCCAAATAATGATTTTATTTTGACTGATAGTGACCTGTTCGTTGCAACAAATTGATGAGCAATGCTTTTTTATAATGCCAACTTTGTATACAAAAGTTG*CACCTGCATACTAAC*GCAAGATGCTAGAGTCTACC*CGCTTCACGCAGGTG*CATGATTACGCCAAGCTATCAACTTTGTATAGAAAAGTTGAACGAGAAACGTAAAATGATATAAATATCAATATATTAAATTAGATTTTGCATAAAAAACAGACTACATAATACTGTAAAACACAACATATCCAGTCACTATGAATCAACTACTTAGATGGTATTAGTGACCTGTACTGCAGGGCGGCCGCATTAGGCACCGACCGAAGAGCAATAATCCT

1. The partial sequence of pLZ-Donor III is color-coded as follows: attL4 in inward orientation in orange, *AtU6-26* promoter in green, two back-to-back *Bsa* I sites marked in a box, optimized gRNA scaffold in blue, *AtU6-26* terminator in grey, and attR3 in outward orientation in red. *Aar*I site *italicized* and underlined with landmark shaded in yellow at specific positions throughout the sequence.

AGGATTATTGCTCTTCGACCGCGTTAACGCTACCATGGAGCTCCAAATAATGATTTTATTTTGACTGATAGTGACCTGTTCGTTGCAACAAATTGATAAGCAATGCTTTTTTATAATGCCAACTTTGTATAGAAAAGTTGTCATTTAGGTGACACTATAGAATACTCAAGCTATGCATCCAACGCG*CACCTGCGTACCGCT*GTCTCTATTGCGACTTGCCTTCCGCACAATACATCATTTCTTCTTAGCTTTTTTTCTTCTTCTTCGTTCATACAGTTTTTTTTTGTTTATCAGCTTACATTTTCTTGAACCGTAGCTTTCGTTTTCTTCTTTTTAACTTTCCATTCGGAGTTTTTGTATCTTGTTTCATAGTTTGTCCCAGGATTAGAATGATTAGGCATCGAACCTTCAAGAATTTGATTGAATAAAACATCTTCATTCTTAAGATATGAAGATAATCTTCAAAAGGCCCCTGGGAATCTGAAAGAAGAGAAGCAGGCCCATTTATATGGGAAAGAACAATAGTATTTCTTATATAGGCCCATTTAAGTTGAAAACAATCTTCAAAAGTCCCACATCGCTTAGATAAGAAAACGAAGCTGAGTTTATATACAGCTAGAGTCGAAGTAGTGATTGAGAGACCGGTCTCAGTTTAAGAGCTATGCTGGAAACAGCATAGCAAGTTTAAATAAGGCTAGTCCGTTATCAACTTGAAAAAGTGGCACCGAGTCGGTGCTTTTTTTTGCAAAATTTTCCAGATCGATTTCTTCTTCCTCTGTTCTTCGGCGTTCAATTTCTGGGGTTTTCTCTTCGTTTTCTGTAACTGAAACCTAAAATTTGACCTAAAAAAAATCTCAAATAATATGATTCAGTGGTTTTGTACTTTTCAGTTAGTTGAGTTTTGCAGTTCCGATGAGATAAACCAATAGTTTA*GAGACACAGCAGGTG*GAATTATCAACTATGTATAATAAAGTTGAACGAGAAACGTAAAATGATATAAATATCAATATATTAAATTAGATTTTGCATAAAAAACAGACTACATAATACTGTAAAACACAACATATCCAGTCACTATGAATCAACTACTTAGATGGTATTAGTGACCTGTACTGCAGGGCGGCCGCATTAGGCACCGACCGAAGAGCAATAATCCT

1. The partial sequence of pLZ-Adaptor III is color-coded as follows: attL4 in inward orientation in orange, stuffer sequence in blue, and attR3 in outward orientation in red. AarI site *italicized* and underlined with landmark shaded in yellow at specific positions throughout the sequence.

AGGATTATTGCTCTTCGACCGCGTTAACGCTACCATGGAGCTCCAAATAATGATTTTATTTTGACTGATAGTGACCTGTTCGTTGCAACAAATTGATAAGCAATGCTTTTTTATAATGCCAACTTTGTATAGAAAAGTTGTCATTTAGGTGACACTATAGAATACTCAAGCTATGCATCCAACGCG*CACCTGCGTACCGCT*GCAAGATGCTAGAGTCTACC*GAGACACAGCAGGTG*GAATTATCAACTATGTATAATAAAGTTGAACGAGAAACGTAAAATGATATAAATATCAATATATTAAATTAGATTTTGCATAAAAAACAGACTACATAATACTGTAAAACACAACATATCCAGTCACTATGAATCAACTACTTAGATGGTATTAGTGACCTGTACTGCAGGGCGGCCGCATTAGGCACCGACCGAAGAGCAATAATCCT

1. The partial sequence of pLZ-Donor IV is color-coded as follows: attL3 in inward orientation in orange, *AtU6-29* promoter in green, two back-to-back *Bsa* I sites marked in a box, optimized gRNA scaffold in blue, *AtU6-29* terminator in grey, and attL2 in inward orientation in red. *Aar*I site *italicized* and underlined with landmark shaded in yellow at specific positions throughout the sequence.

AGGATTATTGCTCTTCGACCCGCTACCATGGAGCTCCAAATAATGATTTTATTTTGACTGATAGTGACCTGTTCGTTGCAACAAATTGATAAGCAATGCTTTTTTATAATGCCAACTTTGTATAATAAAGTTGTTTACTTGTACAGCTCGTCCATGC*CACCTGCGTACGAGA*TTAATCCAAACTACTGCAGCCTGACAGACAAATGAGGATGCAAACAATTTTAAAGTTTATCTAACGCTAGCTGTTTTGTTTCTTCTCTCTGGTGCACCAACGACGGCGTTTTCTCAATCATAAAGAGGCTTGTTTTACTTAAGGCCAATAATGTTGATGGATCGAAAGAAGAGGGCTTTTAATAAACGAGCCCGTTTAAGCTGTAAACGATGTCAAAAACATCCCACATCGTTCAGTTGAAAATAGAAGCTCTGTTTATATATTGGTAGAGTCGACTAAGAGATTGAGAGACCGGTCTCAGTTTAAGAGCTATGCTGGAAACAGCATAGCAAGTTTAAATAAGGCTAGTCCGTTATCAACTTGAAAAAGTGGCACCGAGTCGGTGCTTTTTTTTGGATAGAATTTCCCAGCTTTTTTGCGTGTTTCAGCTCTCATGATCCTTGGCCAATGGGTGTAGTAAATTTTCTGCACATTCATTGGATGGAAAATAATGGTTTTAGCTTTAGGGAATAAGAAAAGTGTATAGGAAGGGGATTTTTGTACAATCACATTTGAATTAGGTCTTTGAAATGACAGGGAATGAGGACATATGATGA*GGTTCACAGCAGGTG*GTCCGTAGCGCGTGCGCCAATTCTGCAGACAAATGGACCCAGCTTTCTTGTACAAAGTTGGCATTATAAGAAAGCATTGCTTATCAATTTGTTGCAACGAACAGGTCACTATCAGTCAAAATAAAATCATTATTTGCCATCCAGCTGCAGGGCGGCCGACCGAAGAGCAATAATCCT

1. The partial sequence of pLZ-Adaptor IV is color-coded as follows: attL3 in inward orientation in orange, stuffer sequence in blue, *AtU6-29* terminator underlined, and attL2 in inward orientation in red. AarI site *italicized* and underlined with landmark shaded in yellow at specific positions throughout the sequence.

AGGATTATTGCTCTTCGACCCGCTACCATGGAGCTCCAAATAATGATTTTATTTTGACTGATAGTGACCTGTTCGTTGCAACAAATTGATAAGCAATGCTTTTTTATAATGCCAACTTTGTATAATAAAGTTGTTTACTTGTACAGCTCGTCCATGC*CACCTGCGTACGAGA*GCAAGATGCTAGAGTCTACC*GGTTCACAGCAGGTG*GTCCGTAGCGCGTGCGCCAATTCTGCAGACAAATGGACCCAGCTTTCTTGTACAAAGTTGGCATTATAAGAAAGCATTGCTTATCAATTTGTTGCAACGAACAGGTCACTATCAGTCAAAATAAAATCATTATTTGCCATCCAGCTGCAGGGCGGCCGACCGAAGAGCAATAATCCT

**Supplemental Sequence Note 3**

**Partial Sequence of eMEMS-*35S* mini pro-Luciferase Reporting System Used in Planta.** The sequence is color-coded as follows: pLZ-Adaptor I in green, underlined; pLZ-Donor II in purple; pLZ-Adaptor III in magenta, underlined; MAS terminator in lowercase letters; HA tag in blue; Luciferase gene in grey; *35S* mini promoter in green; eMEMS in red, which can be replaced with CRMs such as LAP1. gRNA targets are boxed, and numbered in #3, #2, and #1 from top to bottom of the sequence (Figure 3e). Landmarks, residues retained after recognition and cleavage by *Aar*I for Golden Gate assembly, are shaded in yellow at specific positions throughout the sequence.

CTAGGCAAGATGCTAGAGTCTACCTAACTCTCTATTGGACGGTCATTGTTTTAGTTCCACCACGATTATATTTGAAATTTACGTGAGTGTGAGTGAGACTTGCATAAGAAAATAAAATCTTTAGTTGGGAAAAAATTCAATAATATAAATGGGCTTGAGAAGGAAGCGAGGGATAGGCCTTTTTCTAAAATAGGCCCATTTAAGCTATTAACAATCTTCAAAAGTACCACAGCGCTTAGGTAAAGAAAGCAGCTGAGTTTATATATGGTTAGAGACGAAGTAGTGATTGAGAGACCGGTCTCAGTTTAAGAGCTATGCTGGAAACAGCATAGCAAGTTTAAATAAGGCTAGTCCGTTATCAACTTGAAAAAGTGGCACCGAGTCGGTGCTTTTTTTTGGCAAAAATTTTCAGATTTTTTCTTCATCTGTAGATTTCTGGGTTTTTTTTTCCGTTTCGTGAATCATAAGTGAAGTTTTGGATGCAAATCTGCGCGAAAAAAGTTGGACCTGCAATGAGCTTATTTAGATAGCTAAGACAAAGTGATTGGTCCGTTGTTTCGCTGCAAGATGCTAGAGTCTACCGAGAgataatttatttgaaaattcataagaaaagcaaacgttacatgaattgatgaaacaatacaaagacagataaagccacgcacatttaggatattggccgagattactgaatattgagtaagatcacggaatttctgacaggagcatgtcttcaattcagcccaaatggcagttgaaatactcaaaccgccccatatgcaggagcggatcattcattgtttgtttggttgcctttgccaacatgggagtccaagattcTGTATCAACTTCCTCTGCCCTCACCACTTCCAGCGTAATCTGGAACATCGTATGGGTATCTAGATCCAGACAATTTGGACTTTCCGCCCTTCTTGGCCTTTATGAGGATCTCTCTGATTTTTCTTGCGTCGAGTTTTCCGGTAAGACCTTTCGGTACTTCGTCCACAAACACAACTCCTCCGCGCAACTTTTTCGCGGTTGTTACTTGACTGGCGACGTAATCCACGATCTCTTTTTCCGTCATCGTCTTTCCGTGCTCCAAAACAACAACGGCGGCGGGAAGTTCACCGGCGTCATCGTCGGGAAGACCTGCCACGCCCGCGTCGAAGATGTTGGGGTGTTGTAACAATATCGATTCCAATTCAGCGGGGGCCACCTGATATCCTTTGTATTTAATTAAAGACTTCAAGCGGTCAACTATGAAGAAGTGTTCGTCTTCGTCCCAGTAAGCTATGTCTCCAGAATGTAGCCATCCATCCTTGTCAATCAAGGCGTTGGTCGCTTCCGGATTGTTTACATAACCGGACATAATCATAGGTCCTCTGACACATAATTCGCCTCTCTGATTAACGCCCAGCGTTTTCCCGGTATCCAGATCCACAACCTTCGCTTCAAAAAATGGAACAACTTTACCGACCGCGCCCGGTTTATCATCCCCCTCGGGTGTAATCAGAATAGCTGATGTAGTCTCAGTGAGCCCATATCCTTGTCGTATCCCTGGAAGATGGAAGCGTTTTGCAACCGCTTCCCCGACTTCTTTCGAAAGAGGTGCGCCCCCAGAAGCAATTTCGTGTAAATTAGATAAATCGTATTTGTCAATCAGAGTGCTTTTGGCGAAGAATGAAAATAGGGTTGGTACTAGCAACGCACTTTGAATTTTGTAATCCTGAAGGGATCGTAAAAACAGCTCTTCTTCAAATCTATACATTAAGACGACTCGAAATCCACATATCAAATATCCGAGTGTAGTAAACATTCCAAAACCGTGATGGAATGGAACAACACTTAAAATCGCAGTATCCGGAATGATTTGATTGCCAAAAATAGGATCTCTGGCATGCGAGAATCTGACGCAGGCAGTTCTATGCGGAAGGGCCACACCCTTAGGTAACCCAGTAGATCCAGAGGAATTCATTATCAGTGCAATTGTTTTGTCACGATCAAAGGACTCTGGTACAAAATCGTATTCATTAAAACCGGGAGGTAGATGAGATGTGACGAACGTGTACATCGACTGAAATCCCTGGTAATCCGTTTTAGAATCCATGATAATAATTTTCTGGATTATTGGTAATTTTTTTTGCACGTTCAAAATTTTTTGCAACCCCTTTTTGGAAACAAACACTACGGTAGGCTGCGAAATGTTCATACTGTTGAGCAATTCACGTTCATTATAAATGTCGTTCGCGGGCGCAACTGCAACTCCGATAAATAACGCGCCCAACACCGGCATAAAGAATTGAAGAGAGTTTTCACTGCATACGACGATTCTGTGATTTGTATTCAGCCCATATCGTTTCATAGCTTCTGCCAACCGAACGGACATTTCGAAGTATTCCGCGTACGTGATGTTCACCTCGATATGTGCATCTGTAAAAGCAATTGTTCCAGGAACCAGGGCGTATCTCTTCATAGCCTTATGCAGTTGCTCTCCAGCGGTTCCATCCTCTAGAGGATAGAATGGCGCCGGGCCTTTCTTTATGTTTTTGGCGTCTTCCATTGATGCGCCGGGTGTCCTCTCCAAATGAAATGAACTTCCTTATATAGAGGAAGGGTCTTGCGCTGACCAGAGCACGACAGTCTATGTAGGGCGAAAGTTCGTTTGGTTGGCGGGAAAAGTTTTACGGAATTTTATTTTAAAAATAATGATTCTTTTCTACAAAATCTCCTAGACTATGGGAAAGATGATTTAAAAAGTTAATAATATTGTCGTTGTTATCGTCATCGTCATCATCGTCTTTTCTGTTATCTTTTTCTCTTTAAAATTTCGTATTTTTTCTCGTTTACGTAACTATTTAAAATTATATGAACTAACGGTCTCTGTACAGCCGGTTaacc

**Supplemental Sequence Note 4**

**Sequence of dyopCas9-MQ1-Hgy^R^-eMEMS gRNA2 System.** The sequence is color-coded as follows: FLAG in purple; dead yeast-codon optimized Cas9 (dyopCas9) in grey; 3 x NLS in lowercase letters; MQ1 in light blue; P2T in orange; Hygromycin resistant gene in light green; mini *Cyc1* promoter underlined; eMEMS gRNA2 delivering system in magenta. Landmarks, residues retained after recognition and cleavage by *Aar*I for Golden Gate assembly, are shaded in yellow at specific positions throughout the sequence.

AACCATGGATTACAAGGATGACGATGACAAGGGCCGCGGAGACAAGAAGTATTCTATCGGACTGGCCATCGGGACTAATAGCGTCGGGTGGGCCGTGATCACTGACGAGTACAAGGTGCCCTCTAAGAAGTTCAAGGTGCTCGGGAACACCGACCGGCATTCCATCAAGAAAAATCTGATCGGAGCTCTCCTCTTTGATTCAGGGGAGACCGCTGAAGCAACCCGCCTCAAGCGGACTGCTAGACGGCGGTACACCAGGAGGAAGAACCGGATTTGTTACCTTCAAGAGATATTCTCCAACGAAATGGCAAAGGTCGACGACAGCTTCTTCCATAGGCTGGAAGAATCATTCCTCGTGGAAGAGGATAAGAAGCATGAACGGCATCCCATCTTCGGTAATATCGTCGACGAGGTGGCCTATCACGAGAAATACCCAACCATCTACCATCTTCGCAAAAAGCTGGTGGACTCAACCGACAAGGCAGACCTCCGGCTTATCTACCTGGCCCTGGCCCACATGATCAAGTTCAGAGGCCACTTCCTGATCGAGGGCGACCTCAATCCTGACAATAGCGATGTGGATAAACTGTTCATCCAGCTGGTGCAGACTTACAACCAGCTCTTTGAAGAGAACCCCATCAATGCAAGCGGAGTCGATGCCAAGGCCATTCTGTCAGCCCGGCTGTCAAAGAGCCGCAGACTTGAGAATCTTATCGCTCAGCTGCCGGGTGAAAAGAAAAATGGACTGTTCGGGAACCTGATTGCTCTTTCACTTGGGCTGACTCCCAATTTCAAGTCTAATTTCGACCTGGCAGAGGATGCCAAGCTGCAACTGTCCAAGGACACCTATGATGACGATCTCGACAACCTCCTGGCCCAGATCGGTGACCAATACGCCGACCTTTTCCTTGCTGCTAAGAATCTTTCTGACGCCATCCTGCTGTCTGACATTCTCCGCGTGAACACTGAAATCACCAAGGCCCCTCTTTCAGCTTCAATGATTAAGCGGTATGATGAGCACCACCAGGACCTGACCCTGCTTAAGGCACTCGTCCGGCAGCAGCTTCCGGAGAAGTACAAGGAAATCTTCTTTGACCAGTCAAAGAATGGATACGCCGGCTACATCGACGGAGGTGCCTCCCAAGAGGAATTTTATAAGTTTATCAAACCTATCCTTGAGAAGATGGACGGCACCGAAGAGCTCCTCGTGAAACTGAATCGGGAGGATCTGCTGCGGAAGCAGCGCACTTTCGACAATGGGAGCATTCCCCACCAGATCCATCTTGGGGAGCTTCACGCCATCCTTCGGCGCCAAGAGGACTTCTACCCCTTTCTTAAGGACAACAGGGAGAAGATTGAGAAAATTCTCACTTTCCGCATCCCCTACTACGTGGGACCCCTCGCCAGAGGAAATAGCCGGTTTGCTTGGATGACCAGAAAGTCAGAAGAAACTATCACTCCCTGGAACTTCGAAGAGGTGGTGGACAAGGGAGCCAGCGCTCAGTCATTCATCGAACGGATGACTAACTTCGATAAGAACCTCCCCAATGAGAAGGTCCTGCCGAAACATTCCCTGCTCTACGAGTACTTTACCGTGTACAACGAGCTGACCAAGGTGAAATATGTCACCGAAGGGATGAGGAAGCCCGCATTCCTGTCAGGCGAACAAAAGAAGGCAATTGTGGACCTTCTGTTCAAGACCAATAGAAAGGTGACCGTGAAGCAGCTGAAGGAGGACTATTTCAAGAAAATTGAATGCTTCGACTCTGTGGAGATTAGCGGGGTCGAAGATCGGTTCAACGCAAGCCTGGGTACCTACCATGATCTGCTTAAGATCATCAAGGACAAGGATTTTCTGGACAATGAGGAGAACGAGGACATCCTTGAGGACATTGTCCTGACTCTCACTCTGTTCGAGGACCGGGAAATGATCGAGGAGAGGCTTAAGACCTACGCCCATCTGTTCGACGATAAAGTGATGAAGCAACTTAAACGGAGAAGATATACCGGATGGGGACGCCTTAGCCGCAAACTCATCAACGGAATCCGGGACAAACAGAGCGGAAAGACCATTCTTGATTTCCTTAAGAGCGACGGATTCGCTAATCGCAACTTCATGCAACTTATCCATGATGATTCCCTGACCTTTAAGGAGGACATCCAGAAGGCCCAAGTGTCTGGACAAGGTGACTCACTGCACGAGCATATCGCAAATCTGGCTGGTTCACCCGCTATTAAGAAGGGTATTCTCCAGACCGTGAAAGTCGTGGACGAGCTGGTCAAGGTGATGGGTCGCCATAAACCAGAGAACATTGTCATCGAGATGGCCAGGGAAAACCAGACTACCCAGAAGGGACAGAAGAACAGCAGGGAGCGGATGAAAAGAATTGAGGAAGGGATTAAGGAGCTCGGGTCACAGATCCTTAAAGAGCACCCGGTGGAAAACACCCAGCTTCAGAATGAGAAGCTCTATCTGTACTACCTTCAAAATGGACGCGATATGTATGTGGACCAAGAGCTTGATATCAACAGGCTCTCAGACTACGACGTGGACGCCATCGTCCCTCAGAGCTTCCTCAAAGACGACTCAATTGACAATAAGGTGCTGACTCGCTCAGACAAGAACCGGGGAAAGTCAGATAACGTGCCCTCAGAGGAAGTCGTGAAAAAGATGAAGAACTATTGGCGCCAGCTTCTGAACGCAAAGCTGATCACTCAGCGGAAGTTCGACAATCTCACTAAGGCTGAGAGGGGCGGACTGAGCGAACTGGACAAAGCAGGATTCATTAAACGGCAACTTGTGGAGACTCGGCAGATTACTAAACATGTCGCCCAAATCCTTGACTCACGCATGAATACCAAGTACGACGAAAACGACAAACTTATCCGCGAGGTGAAGGTGATTACCCTGAAGTCCAAGCTGGTCAGCGATTTCAGAAAGGACTTTCAATTCTACAAAGTGCGGGAGATCAATAACTATCATCATGCTCATGACGCATATCTGAATGCCGTGGTGGGAACCGCCCTGATCAAGAAGTACCCAAAGCTGGAAAGCGAGTTCGTGTACGGAGACTACAAGGTCTACGACGTGCGCAAGATGATTGCCAAATCTGAGCAGGAGATCGGAAAGGCCACCGCAAAGTACTTCTTCTACAGCAACATCATGAATTTCTTCAAGACCGAAATCACCCTTGCAAACGGTGAGATCCGGAAGAGGCCGCTCATCGAGACTAATGGGGAGACTGGCGAAATCGTGTGGGACAAGGGCAGAGATTTCGCTACCGTGCGCAAAGTGCTTTCTATGCCTCAAGTGAACATCGTGAAGAAAACCGAGGTGCAAACCGGAGGCTTTTCTAAGGAATCAATCCTCCCCAAGCGCAACTCCGACAAGCTCATTGCAAGGAAGAAGGATTGGGACCCTAAGAAGTACGGCGGATTCGATTCACCAACTGTGGCTTATTCTGTCCTGGTCGTGGCTAAGGTGGAAAAAGGAAAGTCTAAGAAGCTCAAGAGCGTGAAGGAACTGCTGGGTATCACCATTATGGAGCGCAGCTCCTTCGAGAAGAACCCAATTGACTTTCTCGAAGCCAAAGGTTACAAGGAAGTCAAGAAGGACCTTATCATCAAGCTCCCAAAGTATAGCCTGTTCGAACTGGAGAATGGGCGGAAGCGGATGCTCGCCTCCGCTGGCGAACTTCAGAAGGGTAATGAGCTGGCTCTCCCCTCCAAGTACGTGAATTTCCTCTACCTTGCAAGCCATTACGAGAAGCTGAAGGGGAGCCCCGAGGACAACGAGCAAAAGCAACTGTTTGTGGAGCAGCATAAGCATTATCTGGACGAGATCATTGAGCAGATTTCCGAGTTTTCTAAACGCGTCATTCTCGCTGATGCCAACCTCGATAAAGTCCTTAGCGCATACAATAAGCACAGAGACAAACCAATTCGGGAGCAGGCTGAGAATATCATCCACCTGTTCACCCTCACCAATCTTGGTGCCCCTGCCGCATTCAAGTACTTCGACACCACCATCGACCGGAAACGCTATACCTCCACCAAAGAAGTGCTGGACGCCACCCTCATCCACCAGAGCATCACCGGACTTTACGAAACTCGGATTGACCTCTCACAGCTCGGAGGGGATGAGGGAGCTGATccaaaaaagaagagaaaggtagatccaaagaagaagagaaaggttgaccccaagaagaagaggaaggtgGCTAGGGACAGCAAAGTGGAGAACAAAACAAAGAAGCTGAGAGTGTTCGAAGCCTTCGCCGGCATTGGCGCCCAGAGAAAGGCCCTGGAGAAAGTGAGGAAGGACGAGTACGAGATCGTGGGACTGGCCGAGTGGTATGTGCCCGCCATCGTCATGTACCAGGCCATCCATAACAACTTCCACACCAAGCTCGAGTACAAGTCCGTCAGCAGAGAGGAGATGATCGACTACCTCGAGAACAAGACCCTGTCCTGGAACAGCAAGAACCCCGTCAGCAACGGATACTGGAAGAGGAAGAAGGATGACGAGCTGAAGATCATCTACAACGCCATCAAGCTGTCCGAAAAGGAGGGCAACATTTTCGACATCAGGGACCTCTACAAGAGGACACTGAAGAACATCGACCTGCTCACCTACAGCTTCCCTAGCCAGGACCTGAGCCAGCTAGGCATCCAGAAGGGCATGAAGAGGGGAAGCGGCACCAGATCCGGCCTGCTCTGGGAGATCGAAAGAGCCCTGGACTCCACCGAGAAGAACGACCTGCCTAAGTACCTCCTCATGGAGAACGTGGGAGCCCTGCTGCACAAGAAGAACGAGGAGGAGCTGAACCAATGGAAGCAGAAGCTGGAGAGCCTGGGCTACCAGAACAGCATCGAAGTCCTCAATGCTGCCGATTTCGGATCCAGCCAGGCCAGGAGGAGAGTGTTCATGATCTCCACCCTCAATGAGTTCGTGGAACTGCCTAAGGGCGACAAGAAGCCCAAGAGCATCAAAAAGGTGCTGAACAAGATCGTGAGCGAGAAGGACATCCTCAACAACCTGCTGAAATACAACCTCACCGAATTCAAGAAGACCAAGTCCAACATCAACAAGGCCAGCCTGATCGGCTACTCCAAGTTCAACTCCGAGGGCTATGTGTACGACCCCGAGTTCACAGGCCCCACACTGACAGCTGCAGGCGCCAACTCCAGGATCAAGATCAAGGACGGCAGCAACATCAGGAAGATGAACAGCGACGAGACCTTCCTGTACATCGGCTTTGACAGCCAGGACGGCAAGAGAGTGAACGAAATCGAGTTCCTGACCGAGAACCAGAAGATCTTCGTGTGTGGCAACAGCATCAGCGTGGAGGTGCTGGAGGCCATCATTGACAAGATCGGCGGCGGAAGCGGAGCTACTAACTTCAGCCTGCTGAAGCAGGCTGGAGACGTGGAGGAGAACCCTGGACCTGGTAGCAAAAAGCCTGAACTCACCGCGACGTCTGTCGAGAAGTTTCTGATCGAAAAGTTCGACAGCGTCTCCGACCTGATGCAGCTCTCGGAGGGCGAAGAATCTCGTGCTTTCAGCTTCGATGTAGGAGGGCGTGGATATGTCCTGCGGGTAAATAGCTGCGCCGATGGTTTCTACAAAGATCGTTATGTTTATCGGCACTTTGCATCGGCCGCGCTCCCGATTCCGGAAGTGCTTGACATTGGGGAATTCAGCGAGAGCCTGACCTATTGCATCTCCCGCCGTGCACAGGGTGTCACGTTGCAAGACCTGCCTGAAACCGAACTGCCCGCTGTTCTGCAGCCGGTCGCGGAGGCCATGGATGCGATCGCTGCGGCCGATCTTAGCCAGACGAGCGGGTTCGGCCCATTCGGACCGCAAGGAATCGGTCAATACACTACATGGCGTGATTTCATATGCGCGATTGCTGATCCCCATGTGTATCACTGGCAAACTGTGATGGACGACACCGTCAGTGCGTCCGTCGCGCAGGCTCTCGATGAGCTGATGCTTTGGGCCGAGGACTGCCCCGAAGTCCGGCACCTCGTGCACGCGGATTTCGGCTCCAACAATGTCCTGACGGACAATGGCCGCATAACAGCGGTCATTGACTGGAGCGAGGCGATGTTCGGGGATTCCCAATACGAGGTCGCCAACATCTTCTTCTGGAGGCCGTGGTTGGCTTGTATGGAGCAGCAGACGCGCTACTTCGAGCGGAGGCATCCGGAGCTTGCAGGATCGCCGCGGCTCCGGGCGTATATGCTCCGCATTGGTCTTGACCAACTCTATCAGAGCTTGGTTGACGGCAATTTCGATGATGCAGCTTGGGCGCAGGGTCGATGCGACGCAATCGTCCGATCCGGAGCCGGGACTGTCGGGCGTACACAAATCGCCCGCAGAAGCGCGGCCGTCTGGACCGATGGCTGTGTAGAAGTACTCGCCGATAGTGGAAACCGACGCCCCAGCACTCGTCCGAGGGCAAAGGAATAGGCTCAGTGCTAACTCGAGTCATGTAATTAGTTATGTCACGCTTACATTCACGCCCTCCCCCCACATCCGCTCTAACCGAAAAGGAAGGAGTTAGACAACCTGAAGTCTAGGTCCCTATTTATTTTTTTATAGTTATGTTAGTATTAAGAACGTTATTTATATTTCAAATTTTTCTTTTTTTTCTGTACAGACGCGTGTACGCATGTAACATTATACTGAAAACCTTGCTTGAGAAGGTTTTGGGACGCTCGAAGGCTTTAATTTGCGGCCGGTACGCAAGATGCTAGAGTCTACCTAAGCCAGCCGGGTAATTCTTTGAAAAGATAATGTATGATTATGCTTTCACTCATATTTATACAGAAACTTGATGTTTTCTTTCGAGTATATACAAGGTGATTACATGTACGTTTGAAGTACAACTCTAGATTTTGTAGTGCCCTCTTGGGCTAGCGGTAAAGGTGCGCATTTTTTCACACCCTACAATGTTCTGTTCAAAAGATTTTGGTCAAACGCTGTAGAAGTGAAAGTTGGTGCGCATGTTTCGGCGTTCGAAACTTCTCCGCAGTGAAAGATAAATGATCTTCTACAAAATCTCCTAGACTATGTTTTAGAGCTAGAAATAGCAAGTTAAAATAAGGCTAGTCCGTTATCAACTTGAAAAAGTGGCACCGAGTCGGTGGTGCTTTTTTTGTTTTTTATGTCTCGGCGAATTGGGTGCGGCCGCGTTTGTCACCTTTCATAGGCCTGACGAAAGGGCCTCGTGGCAAGATGCTAGAGTCTACCCTAG

**Supplemental Sequence Note 5**

**Partial Sequence of Three Donor and Three Adaptor Vectors Used in Yeast, Numbered from A to F.** They directly synthesized as gBlocks gene fragments and inserted into pLZ-sacB via *Sap* I sites using Goden Gate technology.

1. The partial sequence of pLZ-Y-Donor I is color-coded as follows: *SNR52* promoter in light blue, two back-to-back Bsa I sites marked in a box, gRNA scaffold in light green, and *SUP4* terminator in grey. Landmarks, after *Aar*I recognition and cleavage, are shaded in grey at specific positions throughout the sequence.

GTACTCTTTGAAAAGATAATGTATGATTATGCTTTCACTCATATTTATACAGAAACTTGATGTTTTCTTTCGAGTATATACAAGGTGATTACATGTACGTTTGAAGTACAACTCTAGATTTTGTAGTGCCCTCTTGGGCTAGCGGTAAAGGTGCGCATTTTTTCACACCCTACAATGTTCTGTTCAAAAGATTTTGGTCAAACGCTGTAGAAGTGAAAGTTGGTGCGCATGTTTCGGCGTTCGAAACTTCTCCGCAGTGAAAGATAAATGATCAGAGACCGGTCTCAGTTTTAGAGCTAGAAATAGCAAGTTAAAATAAGGCTAGTCCGTTATCAACTTGAAAAAGTGGCACCGAGTCGGTGGTGCTTTTTTTGTTTTTTATGTCTCGGCGAATTGGGTACCGGCCTAAG

1. The partial sequence of pLZ-Y-Adaptor I. Landmarks, after *Aar*I recognition and cleavage, are shaded in grey at specific positions. Between them is stuffer sequence.

GTACGCAAGATGCTAGAGTCTACCTAAG

1. The partial sequence of pLZ-Y-Donor II is color-coded as follows: *SNR52* promoter in light blue, two back-to-back Bsa I sites marked in a box, gRNA scaffold in light green, and *SUP4* terminator in grey. Landmarks, after *Aar*I recognition and cleavage, are shaded in grey at specific positions throughout the sequence.

TAAGTCTTTGAAAAGATAATGTATGATTATGCTTTCACTCATATTTATACAGAAACTTGATGTTTTCTTTCGAGTATATACAAGGTGATTACATGTACGTTTGAAGTACAACTCTAGATTTTGTAGTGCCCTCTTGGGCTAGCGGTAAAGGTGCGCATTTTTTCACACCCTACAATGTTCTGTTCAAAAGATTTTGGTCAAACGCTGTAGAAGTGAAAGTTGGTGCGCATGTTTCGGCGTTCGAAACTTCTCCGCAGTGAAAGATAAATGATCAGAGACCGGTCTCAGTTTTAGAGCTAGAAATAGCAAGTTAAAATAAGGCTAGTCCGTTATCAACTTGAAAAAGTGGCACCGAGTCGGTGGTGCTTTTTTTGTTTTTTATGTCTCGGCGAATTGGGTACCGGCCCGTG

1. The partial sequence of pLZ-Y-Adaptor II. Landmarks, after *Aar*I recognition and cleavage, are shaded in grey at specific positions. Between them is stuffer sequence.

TAAGGCAAGATGCTAGAGTCTACCCGTG

1. The partial sequence of pLZ-Y-Donor III is color-coded as follows: *SNR52* promoter in light blue, two back-to-back Bsa I sites marked in a box, gRNA scaffold in light green, and *SUP4* terminator in grey. Landmarks, after *Aar*I recognition and cleavage, are shaded in grey at specific positions throughout the sequence.

CGTGTCTTTGAAAAGATAATGTATGATTATGCTTTCACTCATATTTATACAGAAACTTGATGTTTTCTTTCGAGTATATACAAGGTGATTACATGTACGTTTGAAGTACAACTCTAGATTTTGTAGTGCCCTCTTGGGCTAGCGGTAAAGGTGCGCATTTTTTCACACCCTACAATGTTCTGTTCAAAAGATTTTGGTCAAACGCTGTAGAAGTGAAAGTTGGTGCGCATGTTTCGGCGTTCGAAACTTCTCCGCAGTGAAAGATAAATGATCAGAGACCGGTCTCAGTTTTAGAGCTAGAAATAGCAAGTTAAAATAAGGCTAGTCCGTTATCAACTTGAAAAAGTGGCACCGAGTCGGTGGTGCTTTTTTTGTTTTTTATGTCTCGGCGAATTGGGTACCGGCCCTAG

1. The partial sequence of pLZ-Y-Adaptor III. Landmarks, after *Aar*I recognition and cleavage, are shaded in grey at specific positions. Between them is stuffer sequence.

CGTGGCAAGATGCTAGAGTCTACCCTAG

**SI References**

1. X. Wang, C. G. Duan, K. Tang et al., “RNA-binding Protein Regulates Plant DNA Methylation by Controlling mRNA Processing at the Intronic Heterochromatin-containing Gene *IBM1*,” *Proceedings of the National Academy of Sciences of the United States of America* (2013) **110**: 15467-15472.

2. M. Lei, H. Zhang, R. Julian et al., “Regulatory Link between DNA Methylation and Active Demethylation in *Arabidopsis*,” *Proceedings of the National Academy of Sciences of the United States of America* (2015) **112**: 3553-3557.

3. B. J. Rauch, M. R. Silvis, J. F. Hultquist et al., “Inhibition of CRISPR-Cas9 with Bacteriophage Proteins,” *Cell* (2017) **168**: 150-158.e110.

4. F. Jiang, J.-J. Liu, B. A. Osuna et al., “Temperature-responsive Competitive Inhibition of CRISPR-Cas9,” *Molecular Cell* (2019) **73**: 601-610.e605.

5. F. Bubeck, M. D. Hoffmann, Z. Harteveld et al., “Engineered anti-CRISPR Proteins for Optogenetic Control of CRISPR-Cas9,” *Nature Methods* (2018) **15**: 924-927.

6. Z. Lang, Y. Wang, K. Tang et al., “Critical Roles of DNA Demethylation in the Activation of Ripening-induced Genes and Inhibition of Ripening-repressed Genes in Tomato Fruit,” *Proceedings of the National Academy of Sciences of the United States of America* (2017) **114**: E4511-E4519.

7. K. W. Earley, J. R. Haag, O. Pontes et al., “Gateway-compatible Vectors for Plant Functional Genomics and pPoteomics,” *The Plant Journal* (2006) **45**: 616-629.
